# Are immigrants allowed to criticize the government? Ingroup identity, economic threat, and majority group support for immigrant civil liberties in the US, Switzerland, and Turkey

**DOI:** 10.3389/fsoc.2025.1520889

**Published:** 2025-05-23

**Authors:** Mia K. Gandenberger, Beyza E. Buyuker, Anita Manatschal, Alexandra Filindra

**Affiliations:** ^1^Swiss Forum for Migration and Population Studies, Université de Neuchâtel, Neuchâtel, Switzerland; ^2^Department of Political Science, University of Illinois Chicago, Chicago, IL, United States

**Keywords:** political intolerance, civil liberties, social identity, economic deprivation, immigration and migration

## Abstract

Assaults on immigrants’ civil liberties have been on the rise across Western countries. This study asks whether majority-group natives exhibit less political tolerance (i.e., support for restrictions on civil rights and liberties) toward immigrants who criticize the government compared to citizens, adding thereby a neglected element to the discussion on the conflicted nexus between migration and citizenship. Drawing on social identity theory and theories of economic threat, we find that across three countries (US, Switzerland, and Turkey) immigrant critics are more strongly penalized. However, the size of the penalty is not moderated by ingroup identity salience, but there is evidence in the US that ingroup victimhood—a different measure of ingroup attitudes—does moderate the treatment effect. Moreover, in all three countries, the treatment effect is amplified by economic threat, and in the US and Turkey, but not in Switzerland, we find significant three-way interactions between the treatment, ingroup identity salience, and economic threat, showing that economic threat activates the effect of ingroup salience. Our findings add to the inconclusive existing evidence on the link between identity salience and political intolerance, by showing that only in combination with realistic feelings of threat (economic threat or victimization) will national or white identity amplify political intolerance towards immigrants.

## Introduction

Do White Americans, German-speaking Swiss and ethnic Turk citizens of their respective countries, that is the groups that form the dominant ethnic group in each state, support restrictions on the civil liberties and civil rights of immigrant critics? Furthermore, do members of these dominant majority groups who exhibit higher attachments to this native majority show greater political intolerance towards immigrant critics and if so, under what conditions? Does economic anxiety, a factor known to animate nativism and xenophobia (Citrin et al., 1997; Filindra and Pearson-Merkowitz, 2013), play a role in expressions of political intolerance towards immigrants?

Traditionally, political tolerance has been defined as extending civil liberties (that is the right to free speech, expression, and assembly) for groups one dislikes, whether because of their political ideology or their social position and standing. This literature has its roots in the American “Red Scare” of the 1950s when the House Committee on Unamerican Activities targeted authors, artists, and activists based on their ideology. From very early on, scholars measured political tolerance in terms of one’s willingness to allow the proponents of ideas one dislikes to not only publicly express their views, for example by making a speech in the community, but as the right not to be excluded because of one’s membership in a disliked ascriptive or ideological group from having their book available at the local library, working as a teacher in high school or university whether they can disseminate their ideas to young people, and being politically active in the form of voting, running for office, or participating in public protests (Gibson and Bingham, 1982; Stouffer, 1955). These forms of political intolerance have come to sharp focus in recent months as the Trump Administration moves to remove books by African American authors such as Maya Angelou from the shelves of military academies, and ties federal funding to Universities’ acceptance of federal audits of the content of classes and research.

Recent events related to the treatment of foreign students, legal permanent residents, and even naturalized citizens who have been deported or threatened with deportation because of their open expression of ideas that the Trump Administration dislikes further underscores the urgency for scholars to revisit the social origins of political (in)tolerance. But political intolerance has not been limited to prominent activists and scholars. Everyday non-citizens have been targeted in various ways.

Springfield, a city of 60,000 struggling to overcome joblessness and the woes that followed the pandemic, is an unlikely place to become the focus of a US presidential campaign. However, the town’s decision in 2014 to invite 20,000 Haitian immigrants to settle there in hopes of generating new jobs and revenue became a lightning rod. The transition was not smooth. Some residents perceived the newcomers as a strain on resources, a driver of higher prices, and a threat to their economic well-being (Bailey, 2024). Others viewed them as unwelcome ethnic “others.” Then, during the 2024 Presidential debate, Donald Trump repeated rumors that Haitian immigrants steal and eat their white American neighbors’ pets. “In Springfield, [t]hey’re eating the pets of the people that live there,” Trump declared to an audience of 67 million people. The message was amplified through social media and rightwing TV programs. Although town leaders and the state’s governor rushed to debunk the story, surveys show that 26% of all Americans and 52% of Trump supporters believed the rumors.^1^

Facing threats of violence, Haitian immigrants in Springfield not only criticized Trump but filed a lawsuit against him (Smith, 2024). In response, Trump doubled down, threatening the immigrants with mass deportations for speaking out—an undemocratic violation of due process rights (Matza, 2024). Many in the American public followed suit: surveys conducted after the Springfield events show that a majority (52%) of Americans and a vast majority of Trump’s followers also support mass deportations.^2^ More recently, the Trump Administration has deported scholars, including legal permanent residents, who criticized Trump’s policies. Foreign visitors have been denied entry for the same reason.

This phenomenon of political intolerance towards immigrants and other outgroups motivated by nativism and a desire to protect the identity of the citizens who make up society’s dominant group is not limited to the US. Across the world, from the Americas to Asia and Europe, there have been crackdowns on the free expression of non-citizen dissenters (Amnesty International, 2024). As in the case of Trump, these phenomena are often accompanied by claims of defending national or ethnic identity, albeit one that defines the political community in ascriptive or exclusive terms. And as in the case of Springfield, many people follow suit, supporting unjust and undemocratic penalties against immigrants. This backlash on immigrants’ right to free speech raises several questions. First, do citizens who belong to the native ethnoracial majority group (the in-group) support restrictions on the civil liberties of immigrant critics (the outgroup) relative to ingroup critics? Second, does ingroup identity salience intensify such a backlash, and under what conditions? By analyzing political intolerance towards immigrants, and thus, the question of who has a say in the imagined political community in people’s view, we study a so far neglected element in the discussion on the conflicted nexus between migration and citizenship.

Political scientists have long argued that loyalties to ascriptive groups may undermine democratic principles (Lijphart, 1977). Scholars of social identity in psychology have demonstrated the centrality of ingroup attachments in human behavior. A key finding is that mere ingroup self-categorization, even absent strong attachments, can contribute to discriminatory behaviors towards outgroups such as immigrants (Brewer, 1999; Tajfel and Turner, 1979). Therefore, group categorization alone may lead to differential affordance of civil liberties to insiders (citizens) and outsiders (immigrants).

In addition, the strength of group attachment among members may further amplify the effect of ingroup categorization, with strong identifiers expressing higher hostility and political intolerance towards outgroups. Scholars have studied the effects of two types of ingroup identities: national and racial. Although national identities may appear more inclusive, and some scholars interpret them in civic terms (Almond and Verba, 1963/2015), studies show that they are often undergirded by ascriptive understandings of the “demos,” the imagined political community (Anderson, 1983/1991; Dawkins and Hanson, 2022; Goodman, 2022; Schildkraut, 2007). Multiple studies show that ingroup identity salience undermines democratic principles. For example, ingroup identity salience correlates with support for exclusionary immigration policies, punitive and militarized social control practices, and justification of political violence—all indicators of weak support of democratic norms in the mass public (Filindra, 2023; Jardina, 2019; Macdonald, 2024; Nava, 2023). Others show that strong ingroup identifiers exhibit higher levels of political tolerance for racists (Davis and Perry, 2020) and lower levels of overall political tolerance (Mužík and Šerek, 2021). However, notable earlier studies from South Africa show null effects of either racial or national identity salience on political tolerance in observational data (Gibson, 2006) and some suggest that ingroup identity salience has prosocial effects (Baldassarri and Grossman, 2013).

For two reasons, the reported null effects should not be taken as the final word on the relationship between ingroup identity strength and political tolerance. First, these studies focused on a single country, South Africa, during a major transition and not specifically on immigrants. The recent uptick in political intolerance toward immigrants in the US but also in other parts of the world warrants closer scrutiny of this phenomenon. Second, scholars have shown that explanations for political intolerance may not hold equally across countries as cultural, institutional, and other contextual factors may moderate the effects (Marquart-Pyatt and Paxton, 2007). In fact, there is good reason to expect that the effect of ingroup identities may not be constant but context-dependent (Abrams and Hogg, 2010). Psychologists have argued that threatening external conditions affect ingroup identity salience in downstream decisions because when threatened, for example, when people experience discrimination or economic decline or lack physical security, they seek to restore control, efficacy, and self-esteem (e.g., Fritsche, 2022).

Scholars of political tolerance have also focused on the role of threat as a driver of political intolerance towards disliked groups (Davis and Silver, 2004; Hutchison and Gibler, 2007; Sullivan and Transue, 1999). Although most studies focus on terrorism’s effect on general political tolerance, there is some evidence that other threats, notably economic anxiety, can increase tolerance toward rightwing extremists (Sinclair et al., 2022). At the same time, immigration and race scholars across several countries show that economic threat motivates majority groups to protect racial hierarchy over democracy and support punitiveness and exclusion of immigrants (Erisen, 2016; Erişen and Erdoğan, 2019; Erisen and Kentmen-Cin, 2016; Filindra et al., 2022; Green et al., 2016; Isaksen et al., 2016; Manunta et al., 2022; Manunta et al., 2024). Scholars suggest that economic threat may activate ingroup favoritism as people seek to restore self-esteem and group status, leading to reactionary responses (Fritsche and Jugert, 2017). Taken together, these studies suggest that the effects of ingroup identity salience on the political tolerance of immigrant critics may be moderated by economic threat.

We test our expectations about the main and moderated effects of social identification and social identity salience in combination with economic threat in three identical experiments conducted in the US, Switzerland, and Turkey, three countries with important cultural and institutional differences. All three are multiethnic states grappling with competing visions of belonging. We consider the US a highly likely or typical case for our theory, with Switzerland and Turkey representing two, albeit very different, hard test cases (Seawright and Gerring, 2008). Due to democratic backsliding (Varieties of Democracy, 2021, 2025), and a majoritarian system of democracy that nurtures deep social and partisan cleavages (Vatter et al., 2020), we expect the US to be a most likely case for intergroup polarization and intolerance towards immigrants. Conversely, intolerance should be lower in stable and consensual Swiss democracy, which cultivates inclusion and appeasement, rather than competition between different societal groups (Vatter et al., 2020). Support for an individual right to criticize the government is low in the electoral autocracy Turkey compared to liberal democracies (Varieties of Democracy, 2021; Wike and Simmons, 2015). We expect that higher base rates of political intolerance among ethnic Turks will lead to ceiling effects, making Turkey our second hard test case. The experiments were conducted with ethnoracial majority group members (i.e., non-Hispanic white Americans, German-speaking Swiss, and ethnic Turks). Respondents were randomly assigned to express their support for restrictions to the civil liberties of either “people” or “immigrants” who are critical of the government.

Our results from two pre-registered hypotheses show that in all three countries, respondents are more politically tolerant of ingroup than immigrant critics of the government, evidence of ingroup bias. Our results on the effect of ingroup identity salience show that neither white (US), nor national identity salience (Switzerland and Turkey) moderate the effect of the treatment, but we find that intolerance of immigrant critics increases significantly as a function of white victimhood beliefs in the US, a measure of threatened ingroup identity. Further in line with this threat argument, we find that economic threat amplifies the treatment effect in all three countries. A significant three-way interaction between the treatment, economic threat, and ingroup identity salience emerges further in the US and Turkey. We also show that among those who experience high economic threat (but not low threat) political intolerance of immigrant critics increases significantly as a function of ingroup identity salience. This triple interaction effect only fails to be significant in Switzerland, which is likely due to the high economic prosperity and low levels of inequality in the country, as well as the fact the right-populist threat discourses around migration center on demographics and much less on the economy.

These findings are important because they offer a response to the puzzling since inconclusive evidence from existing research on the relationship between identity salience and political tolerance. Our results show that only in combination with feelings of realistic threat (economic threat or victimization) will national or white identity amplify political intolerance towards immigrants.

## Social identity

Scholars of comparative politics have long worried about the effect of group attachments on democratic institutions, fearing that ingroup identities can lead to discord and democratic backsliding (Lijphart, 1977). Social identity theory has confirmed that there is reason to worry. Specifically, psychologists have demonstrated that attachment to ingroups can be automatic and deep because it fulfills needs for self-esteem, positive distinctiveness, solidarity, and belonging. Furthermore, even when they harbor no outgroup hostility, ingroup members are likely to favor their groups in ways that produce discriminatory outcomes (Brewer, 1999; Filindra et al., 2024; Tajfel and Turner, 1986). The centrality of ingroup membership is evident in research on immigration and political tolerance. Specifically, citizens exhibit greater political tolerance of immigrants who naturalize than those who do not, and among naturalized citizens those who naturalize to fit in rather than do so for reasons of expediency. These findings suggest that when immigrants express an intent to assimilate and become members of the ingroup, citizens respond by extending more rights to them (Noll et al., 2010; Verkuyten et al., 2023; Verkuyten et al., 2022). The social identity thesis leads to our first hypothesis:

*H1:* Majority-group members will be more politically intolerant of immigrants who criticize the government than of people in general voicing government critique.

## Social identity salience

Scholars have also demonstrated that people harbor different levels of attachment to ingroups, and high identifiers may behave more punitively toward outgroups than low identifiers. Ingroup identity salience, national or racial, correlates with support for undemocratic leaders and extreme parties, endorsement of harsh social control policies, increased xenophobia, and justification of political violence (Bai, 2020; Filindra, 2023; Indelicato and Martín, 2024; Jardina, 2019). However, others show null or positive effects of ingroup identity salience on trust in institutions and support for antidemocratic leaders which suggests that ingroup identities have a more complex effect on beliefs and attitudes (Buyuker et al., 2021; Filindra et al., 2023b; Fording and Schram, 2023). Others demonstrate that ingroup identity salience, variously measured, contributes to prosocial behaviors (Baldassarri and Grossman, 2013; Marinthe et al., 2022).

Although early studies assumed that national identities in liberal democracies were based on civic nationalism (Almond and Verba, 1963/2015), more recent studies have shown that such identities hide deep divisions in how people define the “demos,” the imagined political community that is entitled to democratic rights and protections. Specifically, scholars have argued that many people harbor ethnoracial definitions of the political community (i.e., when deciding what makes one a member of the nation, they privilege ascriptive characteristics such as race, ethnicity and religion). Furthermore, those who have strong priors are more likely to support undemocratic leaders, parties, and policies (Buyuker et al., 2023; Dawkins and Hanson, 2022; Filindra, 2023; Goodman, 2022; Schildkraut, 2007).

However, the results are mixed when it comes to the relationship between ingroup identity salience and support for political tolerance. Studies conducted in South Africa indicate that neither national nor racial identity salience is a significant predictor of political tolerance (Gibson, 2006; Gibson and Gouws, 2002). In the US, there is some evidence that white nationalism correlates with greater tolerance of racists (Davis and Perry, 2020). Also, a recent study shows that national identity correlated with reduced political tolerance in the Czech Republic (Mužík and Šerek, 2021). Scholars have not studied the role of ingroup identity salience on the political tolerance of immigrants.

*H2:* Majority-group members who score higher on ingroup identity will be more politically intolerant of immigrants who criticize the government than of people in general voicing government critique.

## The moderating effects of threat

Yet, the mixed evidence on the link between social identity and political tolerance in a variety of contexts and towards different target groups suggests that the relationship is not automatic, but conditional. Threat as a predisposition and as a situational factor is central to political tolerance judgments. Early studies focused on predispositions, as when people tend to view the world as a dangerous place (Altemeyer, 1988), or conceptualized threat as behaviors that violate group norms, such as perceptions that outgroups are violent and dangerous (Sullivan et al., 1982). In this vein, studies show that people are more tolerant of disliked groups that are peaceful than those associated with violence (Petersen et al., 2011). Scholars have also conceived of threat as an environmental or contextual condition that influences attitudes about political tolerance (Marcus et al., 1995).

Others have shown that a threatening context can produce a backlash on citizens’ support for extending rights to outgroups (e.g., Gibson and Gouws, 2002; Sullivan and Transue, 1999). Studies further suggest that uncertainty and unsettled feelings associated with external threats can produce greater political intolerance (Haas and Cunningham, 2014). There is evidence that actual or perceived threats associated with territorial boundaries, terrorism, or the economy can reduce political tolerance for disliked outgroups (Davis and Silver, 2004; Hetherington and Suhay, 2011; Hutchison and Gibler, 2007; Peffley et al., 2015). Furthermore, one study shows that security threat decreases political tolerance for Muslims (Noll et al., 2010). Scholars document that economic threat increases political tolerance for the extreme right (Sinclair et al., 2022). The relationship between various forms of threat and intolerance has been validated across several countries (Erişen and Erdoğan, 2019; Hutchison and Gibler, 2007).

Social identity scholars have long shown that identities are not perennially salient. Instead, the context is crucial for the activation of social identities (Abrams and Hogg, 2010; Ma and Seate, 2017). When identities are threatened because of situational or contextual factors, even when the situation is not directly personally relevant, people experience negative emotions and respond in ways that seek to restore the damage to their positive identity (Tajfel and Turner, 1986). Furthermore, economic threat can affect people’s sense of control and their individual or group-level esteem (Fiske, 2010). Under such conditions, people shift automatically to a collective definition of the self which increases the salience of ingroup identity on downstream decisions (Fritsche, 2022; Fritsche et al., 2013; Stollberg et al., 2017).

Immigration scholars have also shown that economic threat influences majority ingroup members’ support for restrictive and punitive policies and even for undemocratic norms as members of the ingroup majority strive to protect group privileges and their high place on the social stratification system (Erisen, 2016; Erişen and Erdoğan, 2019; Erisen and Kentmen-Cin, 2016; Filindra et al., 2022; Green et al., 2016; Isaksen et al., 2016; Manunta et al., 2022; Manunta et al., 2024). However, these studies have not examined how economic threat may affect the political tolerance of immigrants by moderating the salience of ingroup identities. Our third hypothesis qualifies therefore the previously posited positive relationship between national identity and intolerance (H2), accounting for its conditionality on economic threat:

*H3*: Majority-group members who score higher on ingroup identity will be more politically intolerant of immigrant critics than of people who criticize the government if perceived economic threat is high.

## Data, case selection, and methods

We fielded three identical, pre-registered, survey experiments between January and April 2021 in the US, Switzerland, and Turkey.^3^ We selected these countries for several reasons (see Table 1). All three are multiethnic states that are grappling with ethnocultural and multicultural visions of belonging (Erisen, 2016; Goodman, 2022; Kaya et al., 2020; Schildkraut, 2011). The US and Switzerland are both liberal democracies with strong protections for freedom of speech. However, while the US represents a majoritarian system nurturing deep social and partisan cleavages, Switzerland is a consensus-based democracy, which cultivates inclusion and appeasement, rather than competition, between diverse societal groups (Bernauer and Vatter, 2019). Due to recent democratic backsliding (Varieties of Democracy, 2021, 2025), and a majoritarian system that encourages intergroup polarization, we expect the US to be a most likely case for political intolerance towards immigrants. As one of the most consensual democracies in the world (Vatter, 2008) and a country with lower levels of income inequality than the other two, that has, unlike the other two countries, not experienced episodes of democratic backsliding in recent years, Switzerland, serves as our first hard test case (Manatschal and Bernauer, 2016).^4^ We expect political intolerance towards outgroups to be less pronounced here than in the US.

**Table 1:** Case similarities and differences.

	US	Switzerland	Turkey
Multiethnic	Yes	Yes	Yes
Immigrant-receiving	Yes	Yes	Yes
Liberal democracy	Yes	Yes	No [Electoral autocracy]
Democratic backsliding	Yes	No	Yes
Democratic power sharing	Majoritarian	Consensus	Neither
Support for civil liberties	High	High	Low
Unemployment	Low	Low	High
Economic inequality	High	Middle	High

Turkey is categorized as an electoral autocracy where rights of expression are curtailed for all groups, including citizens (Boese-Schlosser et al., 2022). Democratic backsliding started with the accession of the Justice and Development Party (AKP) to power in 2002, when tutelary democracy started to cede to a competitive authoritarian regime (Esen and Gumuscu, 2016). Support for an individual right to criticize the government is low in Turkey compared to liberal democracies (Wike and Simmons, 2015). We expect thus less room to induce greater political intolerance, making Turkey our second hard case to test our theory.

**Figure 1:**
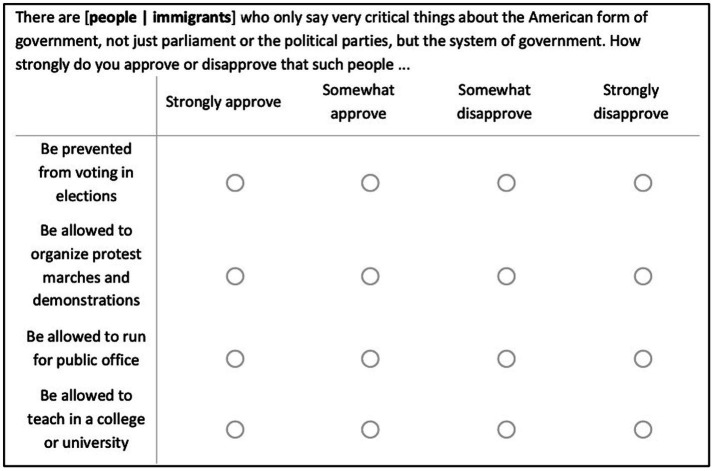
Experimental stimuli on political tolerance (US Version).

We sampled majority-group members only, that is, non-Hispanic White US Americans (*N* = 6,762), native-born German-speaking Swiss (*N* = 2,392), and Turkish-speaking citizens of Turkey (*N* = 2,826).^5^ Respondents were first asked to complete a battery of demographic and attitudinal questions. Subsequently, they were randomly assigned to one of two experimental conditions: a “people critics” of the government” (control) condition or an “immigrant critics” (treatment) condition. We expect that respondents interpret “people” from an ingroup perspective, as members of a group they identify with.^6^ Respondents were then asked to indicate how much they (dis-)approve the respective group should be able to vote, run for office, protest, or teach at a college or university.^7^ The exact experimental manipulation and the four items can be found in Figure 1. We framed the question around criticism of government because that is a key way in which people exercise their civil liberties. (Dis)agreement with these four items specified above measure respondents’ (in)tolerance, meaning their willingness to allow (or restrict) others (and especially disliked others) to freely exercise their civil liberties and rights. Based on these four items, we created an additive index (using equal weights) for political intolerance (αUS = 0.77; αCH = 0.62; αTR = 0.58) where higher values indicate higher intolerance, which serves as our dependent variable.^8^ All variables are rescaled on a 0–1 range.

Our key independent variables are (1) a measure of white identity salience and a measure of white victimhood, which expresses threatened ingroup identity (modeled separately as alternatives) in the US, (2) a measure of national identity salience in Switzerland and Turkey (all on 5-point importance scales), and (3) a measure of economic threat that asks, “How financially well off do you consider yourself to be compared to people like you 30 years ago?” (5-point scale from much worse to much better).^9^ To facilitate the interpretation of 3-way interactions, our figures collapse economic threat into three categories (high, medium, and low).

We opted for national identity in the case of Switzerland and Turkey because racial identity is not directly relevant there, and we had no clear theoretical reason to focus on a regional or other identity. Furthermore, given that many majority group members tend to associate their group with the national identity, we expect that in these contexts, national identity functions similarly to White identity in the US. Our control variables are gender, age, income, education, partisanship, ideology, and authoritarianism. All variables are rescaled on 0–1 scales consistent with the original nature of the variable so that our coefficients can be interpreted as maximum effects. For more details on all variables, question wordings, coding and operationalizations see Appendix B. Summary statistics, histograms and descriptives of dependent variables and key moderators, bivariate correlations and balance tables can be found in Appendices C–G.

For our multivariate analyses, we estimate robust regression models (Verardi and Croux, 2009) controlling for respondent gender, age, education, income, partisanship, ideology, and authoritarianism in all interaction models. The controls are necessary because our interaction measures are not experimentally manipulated (Kam and Trussler, 2017).^10^

## Results

Consistent with our hypotheses, we present three sets of results. First, we show the main treatment effects, then we move to interactions with ingroup identity, and finally, we show three-way interactions between the treatment, ingroup identity, and economic threat. Since our dependent variable is continuous, we use robust linear regression models (Verardi and Croux, 2009).

### Main treatment effects

**Figure 2:**
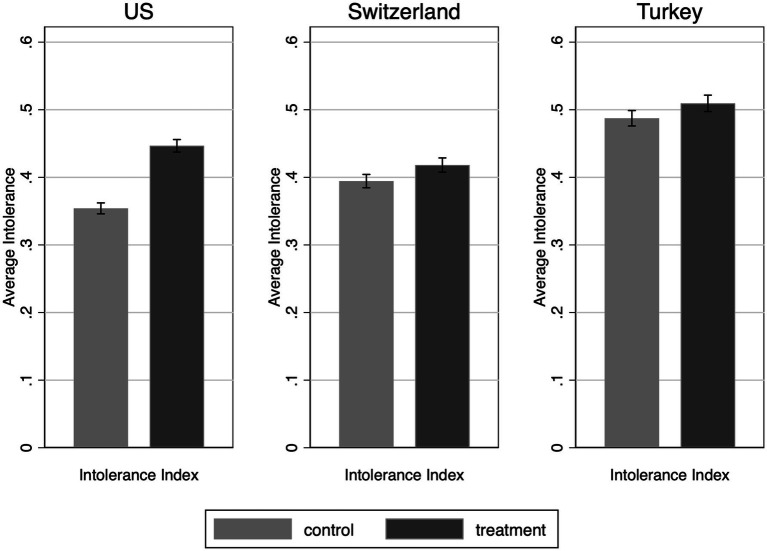
Average treatment effects (ATE) of the immigrant priming treatment on political intolerance for the US, Switzerland, and Turkey.

First, we test H1, which specifies that we should expect higher political intolerance for immigrants due to ingroup favorability. In the US, the political intolerance measure has a mean of 0.400 (SD = 0.262), like Switzerland (Mean = 0.406; SD = 180), but in Turkey average political intolerance is about 10 ppts higher (Mean = 0.498; SD = 0.226). Therefore, our data suggest that white Americans and Swiss Germans are substantially more tolerant than majority group members in Turkey. However, average intolerance scores do not necessarily mean that people are equally politically (in)tolerant of ingroup and immigrant critics of the government. To compute average treatment effects, we use two-sample t-tests (Figure 2). In line with our expectations, respondents are more intolerant of immigrant critics than non-immigrant critics characterized as “people.” This difference is statistically significant in all three countries, but the effect is much larger in the US than elsewhere. Specifically, in the US, the difference between the two groups is almost 15 ppts (*t* = 14.731, *p* < 0.001), while in Switzerland, it is 3 ppts (*t* = 3.225; *p* < 0.001) and in Turkey 2.5 ppts (*t* = 2.591, *p* < 0.01). In the US, the results hold under all model specifications with little change in the magnitude of the effect, but this is not the case elsewhere (see Appendix H for regression results on main effects). Overall, these results show the higher polarization in the US. All three countries are very diverse in population, but in the US, the emotional distance in perception between “the people” and the immigrants is very large among white people which is not the case in the other two countries. Although the Turkish population exhibits higher levels of intolerance on average, this is not because they penalize immigrants at much higher levels.

### Interactions with ingroup identity salience

As a next step, we investigate whether ingroup identity salience moderates the effect of the treatment on political intolerance. The full models are in Appendix I. White identity salience is normally distributed in the US (Mean = 0.52, SD = 0.284), and the same is true for White victimhood (Mean = 0.52, SD = 0.239). However, national identity salience in Switzerland is skewed toward higher levels, with very few people saying that being Swiss is not important to their identity (Mean = 0.719, SD = 0.197). The same is true for Turkey (Mean = 0.838, SD = 0.207). This low variance in Switzerland and Turkey makes it harder to find significant interaction effects.

**Figure 3:**
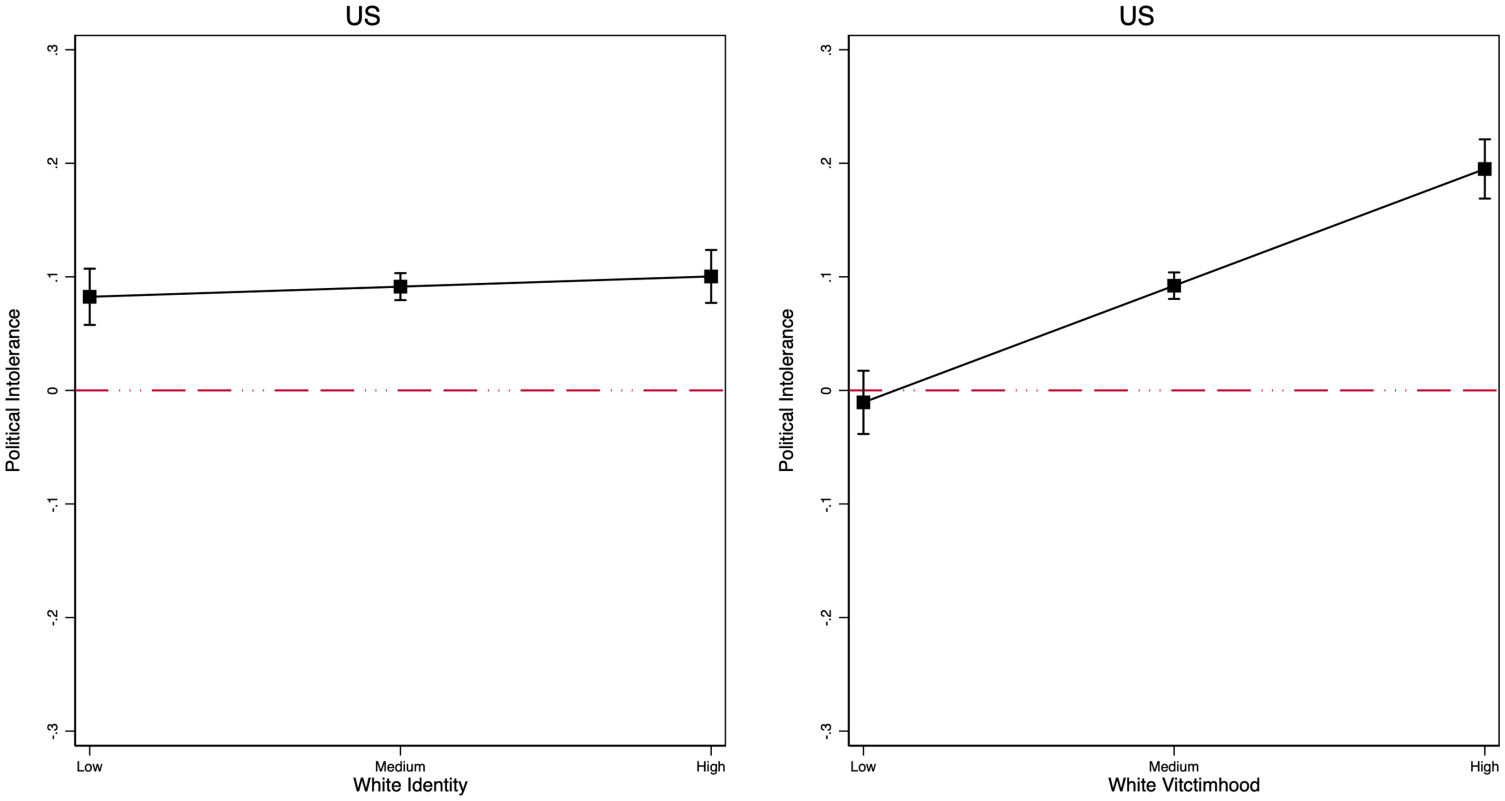
US results (marginal effects) of the interaction between the immigrant treatment and white identity **(left)** and white victimhood **(right)**. Robust regression results, non-Hispanic white people only. Controls include gender, age, education, income, partisanship, ideology, and authoritarianism.

**Figure 4:**
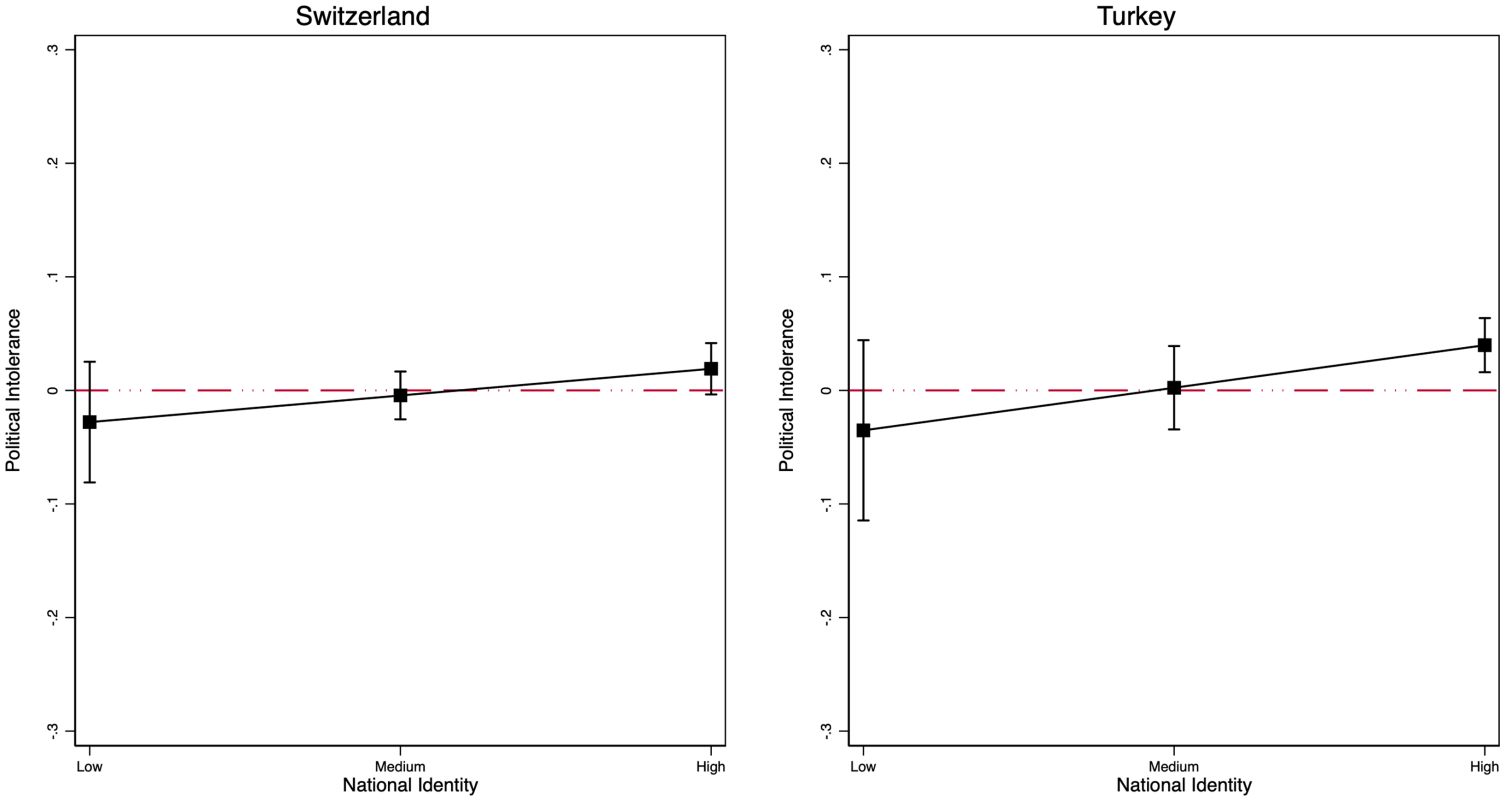
Marginal effects of the interaction between the immigrant treatment and national identity salience in Switzerland **(left)** and Turkey **(right)**. Robust regression results. Respondents are native majority group members only. Controls include gender, age, education, income, partisanship, ideology, and authoritarianism.

As a reminder, theory suggests that identity salience (measured at the national or group level) should magnify intolerance, but some studies that do not focus on immigrants report null effects (Gibson, 2006; Gibson and Gouws, 2002). We test this hypothesis by specifying interaction models with controls. As Figure 3 shows, the interaction is null in the US, where we use white identity as our moderator, and in Switzerland and Turkey (Figure 4), where we interact with national identity salience. Overall, we conclude that ingroup identity salience alone is insufficient to drive political intolerance toward immigrant critics. The same is not the case, however, for white victimhood, a measure that taps into ingroup grievances and imbues identity with threat. White victimhood measures reflect, in part, perceptions of ingroup discrimination and, therefore, can be viewed as a measure of ingroup threat, not simply a measure of ingroup attachment. Specifically, the results show a positive and significant interaction between the treatment and white victimhood (*b* = 0.206; *p* < 0.001). As Figure 3 shows, political intolerance for immigrant critics is about 20 ppts higher among white Americans who score high on victimhood than those at the lowest point of the scale.

To put it another way, respondents at the low end of the victimhood scale do not differentiate between “people” and “immigrants” in the way they respond, whereas those at the top of the scale are significantly more likely to express political intolerance towards immigrant critics than people in general.

Our results are consistent with other studies that utilize ingroup identity salience measures and present null results (Gibson and Gouws, 2002). This, in conjunction with the significant interaction effect of ingroup victimhood, gives credence to the expectation that threat may be necessary to activate ingroup identities and allow them to influence downstream perceptions. However, our study does not include measures of victimhood for all three countries; therefore, we cannot perform identical tests in Switzerland and Turkey.

### Economic threat and identity salience

So far, our analyses suggest that at least one form of threat, perceptions of group victimhood, should drive political intolerance against immigrants. This is consistent with the broader contention in the literature that threat correlates with political intolerance. We will now test, if this applies also to economic threat, which stands in the focus of our theoretical argument (H3).

**Figure 5:**
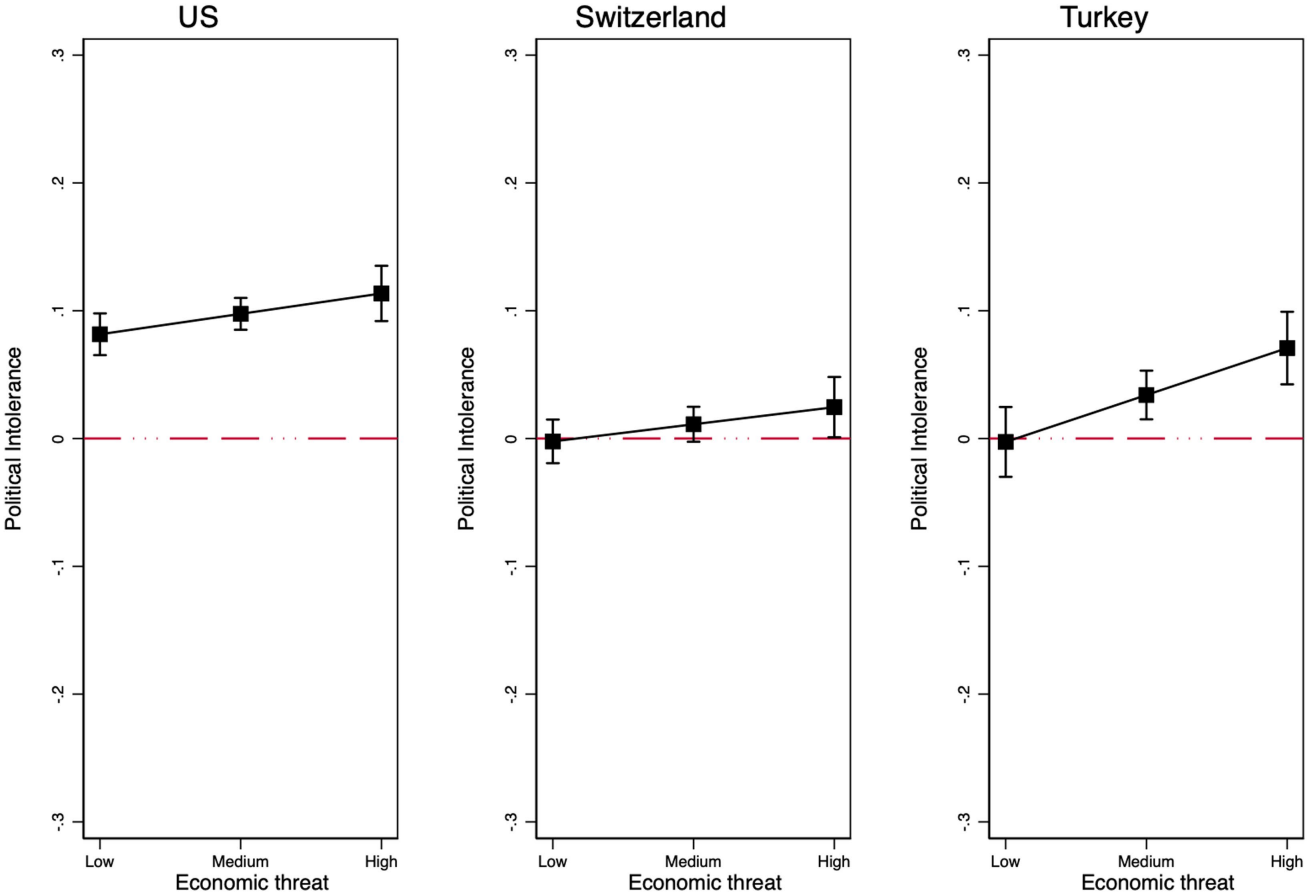
Marginal effects of the interaction between the immigrant treatment and economic threat in the US **(left)**, Switzerland **(center)**, and Turkey **(right)**. Robust regression results. Respondents are native majority group members only. Controls include gender, age, education, income, partisanship, ideology, and authoritarianism.

First, as shown in Appendix Tables I1–I3, the interaction between the treatment and economic threat is positive and statistically significant in all three countries (US: *b* = 0.032; *p* < 0.05; CH: *b* = 0.027; *p* < 0.1; TR: *b* = 0.073; *p* < 0.01, see Figure 5). Across all countries, economic threat increases political intolerance toward immigrant critics between 3–7 ppts. Furthermore, robustness checks show that the interaction remains significant across multiple model specifications (see Appendix J). These results show that political tolerance of immigrant critics is, in part, a function of economic threat across very different societies.

As we noted earlier, economic threat has been shown to influence attitudes through identity salience (Fritsche, 2022; Fritsche et al., 2013; Stollberg et al., 2017). Therefore, it is likely that high ingroup identifiers become intolerant of immigrant critics only under conditions of economic threat. We test this conditional hypothesis using three-way interaction models. Our large sample sizes, especially in the US, allow for this additional level of interaction. The full models are presented in Appendix I.

The three-way interaction is significant in the US and Turkey (US: *b* = 0.171; *p* = 0.001; TR; *b* = 0.245; *p* = 0.016). In both countries, economic threat activates the effect of identity salience on political intolerance towards immigrants. In the US, we further find a significant three-way interaction between the treatment, economic threat, and white victimhood (*b* = 0.111; *p* < 0.05, one-tailed), which suggests that economic threat activates and amplifies the effect not only of identity salience but also of identity threat on political tolerance (Appendix Table I1). However, in Switzerland, national identity and economic threat operate independently and do not amplify one another.

**Figure 6:**
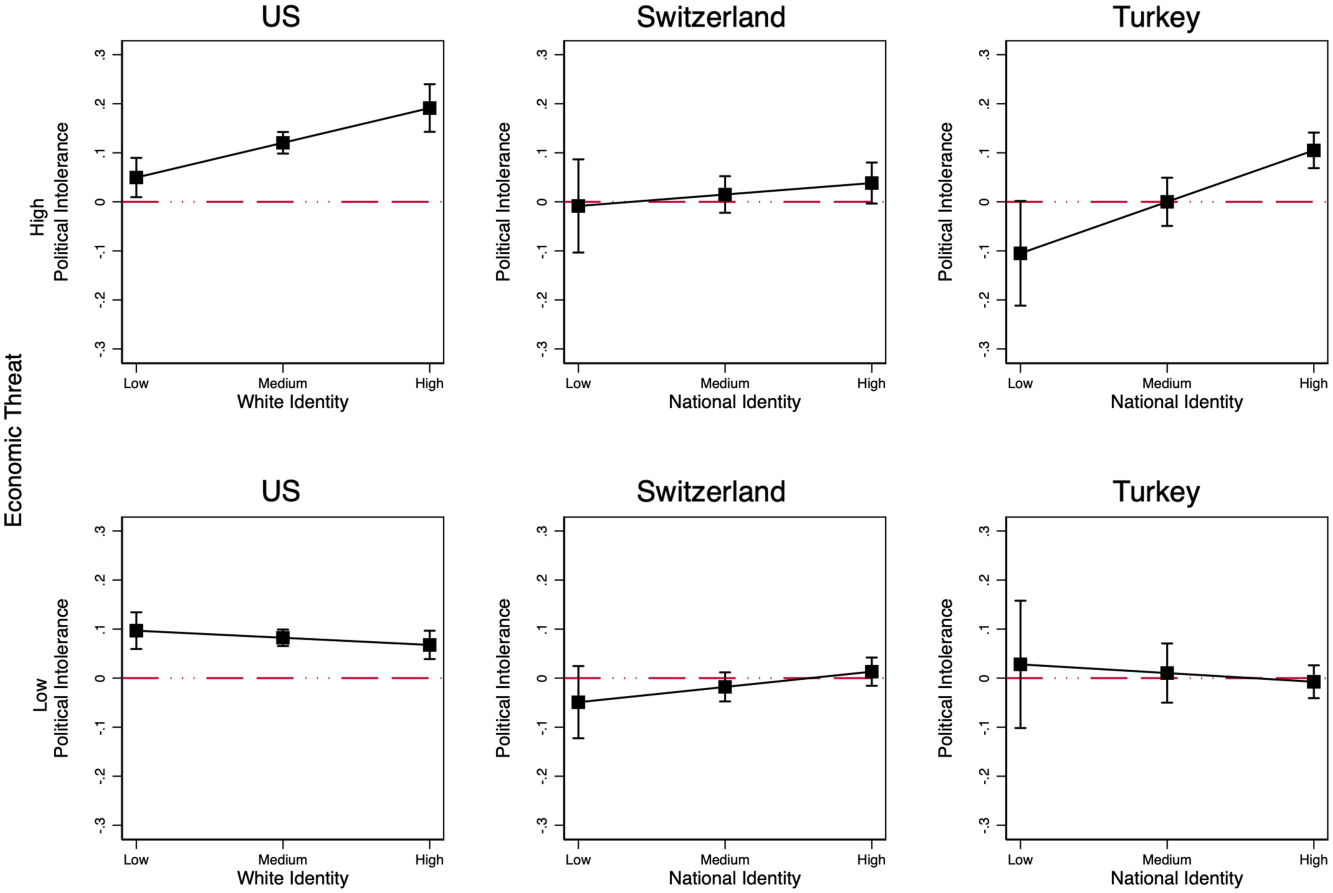
Marginal effects of the three-way interaction between the immigrant treatment, ingroup identity salience, and economic threat (high [**upper panel**] and low [**lower panel**]). Robust regression results, ingroup members only. Controls include gender, age, education, income, partisanship, ideology, and authoritarianism.

Because three-way interaction results are not easy to interpret intuitively, we divided the threat variable into three groups: high, medium, and low. We expect that the effect of the treatment will be a function of ingroup identity salience for respondents who experience high levels of economic threat but not those in the low-threat group. Figure 6 shows the results for the high economic threat group (top panel) and for the low threat group (bottom panel). In the US, among respondents who express high economic threat, political intolerance toward immigrant critics is 12.5 ppts higher for those high on white identity compared to those who score low (*b* = 0.125; *p* = 0.008). Furthermore, among the same group, political intolerance toward immigrants increases by 26 ppts for those high on white victimhood compared to those low on that scale (*b* = 0.265; *p* < 0.001). By contrast, among respondents who show high levels of economic threat in Turkey, political intolerance shifts by 18 ppts when we move from the lowest to the highest level of national identity salience (b = 0.183; *p* = 0.020). For all three countries, the effect of ingroup identity salience on political intolerance for immigrant critics is not significant for people in the low economic threat condition (Figure 6, bottom panel). We take this to mean that the effect of ingroup identity salience is more likely to emerge under high-threat conditions.

### Alternative explanations

Our study privileges ingroup identity and economic threat as key drivers of political intolerance of immigrant critics, but we also need to account for other explanations and reassure the reader that our results hold even when these factors are taken into consideration. This is especially important given the use of interaction models which are not fully causally identified (Kam and Trussler, 2017). Scholars have long attributed political intolerance to authoritarianism and ideology, suggesting that those who score high on authoritarian personality and conservatism are more likely to be politically intolerant towards outgroups (Stenner, 2005; Sullivan and Transue, 1999).

First, when it comes to authoritarianism, we find that in the US, the interaction between authoritarianism and the treatment is positive and significant (*b* = 0.061; *p* < 0.001) but including it in the model does not affect the significance of the interaction with the economic threat measure (for the US see Appendix Tables J1 and Appendix Table J2). Moreover, it does not alter the importance of our three-way interaction between the treatment, identity salience and economic threat. In Switzerland, the interaction with authoritarianism is null across multiple model specifications, and inclusion or exclusion of the measure does not affect our results (see Appendix Table J3). The same is true for Turkey, where the interaction with authoritarianism is mostly null, notably also in the three-way interaction model (see Appendix Table J4). Unlike the expectations of other scholars (Stenner, 2005), authoritarianism does not seem to have consistent effects in all sociopolitical contexts, but polarization matters for its activation. For our purposes, suffice to say that it does not account for the results that we observe in our data.

Second, we tested if interactions of our treatment with political ideology will alter our results. This is because a large literature suggests that ideology is correlated with undemocratic beliefs and responses in the public (e.g., Badaan et al., 2023; Graham and Svolik, 2020). We use political ideology instead of party identity for these additional robustness checks, because it is a comparable measure across all countries (all models control for party identity). The respective interaction is positive and significant in the US (*b* = 0.152, *p* < 0.001). This indicates that conservatives are more likely to be politically intolerant of immigrants than are liberals. However, including this interaction in the model does not change our results, specifically the significant interactions between the treatment and white nationality or victimhood and the treatment and economic threat. Both remain significant and substantively the same. The same is the case for our three-way interaction. Neither in Switzerland nor in Turkey is the treatment effect significantly amplified by ideology, reflecting the lower levels of polarization in both societies. Its inclusion in the model does not change the results of our three-way interactions in these two countries (Appendix J). These findings offer further reassurance that our expectations hold against other possible explanations.

## Discussion and conclusion

Our study set out to investigate the dynamics of political intolerance and the contested borders of the political community by comparing reactions among the majority population to “people” in general and “immigrants” voicing critique of the government. Specifically, we hypothesized that citizens who are members of the native majority group may be less politically tolerant of immigrants and more politically tolerant of members of their own group. Also, we posited that citizens’ strength of attachment to their ingroup may amplify this intolerance. Furthermore, we expected that the effect of citizens’ ingroup identity on political intolerance of immigrant critics would be stronger among those who expressed high levels of economic threat.

We find broad support for our first and third hypothesis, with some notable variance across countries. First, across all three countries, we find a backlash against the civil liberties of immigrant critics. This effect is most pronounced in the US, and, as expected, much weaker in Switzerland and Turkey. Although political intolerance appears to be as high in the US as in Switzerland (but higher in Turkey), polarization along nativity lines is only very high in the US because of Donald Trump’s exceptionally hostile rhetoric, and this is likely a cause of the backlash we see in our study. Although Switzerland is also home to xenophobic rhetoric, the country’s lower levels of polarization and consensual style of politics may dampen this effect. The relatively low backlash against immigrants compared to “people” critics in Turkey may, to some extent, be a consequence of the ceiling effects of political intolerance, which is generally high regardless of the critic’s individual background. Like the situation in the US, a timelier explanation relates to the rhetoric used by Erdogan regarding immigrants, which may influence public opinion on the issue, although in the opposite direction. The Turkish president has traditionally adopted a pro-immigrant and welcoming stance towards the 3.6 million Syrian refugees hosted in Turkey (Morgül, 2023), and he can count on their firm support. Even if, influenced by the increasingly xenophobic campaigns of his competitors, he adopted a more restrictive tone towards Syrians in the recent presidential election campaign, Syrian refugees were relieved by his re-election in spring 2024.^11^

Second, our results show that ingroup identity salience, racial (in the US) or national (Switzerland and Turkey), does not amplify the effect of the treatment on political intolerance. These null results of ingroup identity salience confirm the conclusions of earlier studies showing that there is no significant relationship between ingroup identity and political tolerance (Gibson, 2006). They are also consistent with studies of white identity suggesting prosocial effects in certain contexts even if white identity correlates with conservative positions overall (Filindra et al., 2023a). However, our tests of white victimhood in the US show that when identity is combined with strong emotions, such as feelings of grievance, it does contribute significantly to the backlash against immigrants’ civil liberties. As others have shown, ingroup victimhood beliefs can undermine support for democratic principles—such as civil liberties, but not alone—in the public (Armaly et al., 2022; Filindra et al., 2023a; Filindra et al., 2023b; Zimmerman, 2022). In line with this threat narrative, and hardly surprising, additional analyses reported in the Appendix show that racism and xenophobia also amplify political intolerance towards immigrant critics in all three countries.

Finally, and building further on this threat argument, our results show that economic threat can be an activator of ingroup identity and contribute to the backlash against immigrants’ civil liberties. Specifically, we find that in both the US and Turkey, when economic threat is high, strong ingroup identifiers show higher levels of political intolerance against immigrant critics. This is not the case for conditions of low economic threat where the results are null. Only in Switzerland perceived economic threat fails to have the same activating effect on ingroup identity. Puzzling at first sight, this result is however in line with earlier studies showing that measures of observed economic threat, such as unemployment rates, do not increase individual threat perceptions and are also unrelated to radical right voting propensity in Switzerland (Green et al., 2016). In a similar vein, low skilled workers are not more xenophobic than medium skilled workers in the country (Pecoraro and Ruedin, 2016). Apparently, Switzerland’s high economic welfare and the fact that politicization of migration does not center on the economy, but on demographics and the perceived risk of “over-foreignization” (Manatschal, 2023), explains why economic concerns are not perceived as a salient threat in the country and fail to activate ingroup identity salience. Here it is important to note that the large sample sizes that we employ across all countries and more so in the US make us confident in the reliability of our results. We also show that alternative explanations, such as authoritarianism or political ideology, do not meaningfully alter our results when relevant measures are included in the models.

Our nuanced findings allow us to draw conclusions about the contextual scope conditions of our theory. In line with our expectations, findings on the main experimental effects are weaker in Switzerland. We believe that this is linked to the country’s consensual political context, which effectively tames polarization since tolerance and representation of differing opinions is institutionally guaranteed, e.g., by proportional electoral systems and broad coalition governments on all levels of government. Institutional measures such as consensual power sharing can thus be an efficient guardrail against polarization in political intolerance.

Turkey, in turn, is a hard test case for its autocratic regime, which results in overall elevated levels of political intolerance. In spite of these ceiling effects and president Erdogan’s traditionally inclusive rhetoric towards immigrants, especially Syrian refugees, we find significant main and heterogeneous treatment effects in Turkey in line with our arguments. The fact that we find evidence supporting our main argument, even in the two hard test cases Switzerland and Turkey, though it is clearly weaker, shows that the issue of political intolerance towards immigrants is not unique to the US, but a more general challenge faced by contemporary societies.

Our study advances research on the conflicted migration-citizenship nexus by uncovering international dynamics of exclusion in the realm of immigrants’ civil liberties. However, the documented backlash against immigrants’ right to express dissent has implications that go beyond questions of migration or citizenship status. Tolerance, the agreement to disagree, is a fundamental pillar of democratic governance, distinguishing democracies from authoritarian or totalitarian regimes. The backlash against immigrants’ civil liberties undermines therefore also a core principle of democratic governance.

The study is not without limitations. One could contend that the immigrant prime we use in our treatment is a rather vague concept, and that study participants may associate very different kinds of people - individuals with varying migration trajectories, socio-economic or legal statuses - with this term. Indeed, respondents are likely to react differently depending on whether they are thinking about a highly skilled expat or an irregular migrant voicing the critique. At the same time, research shows that people have rather uniform associations with this term. Not least due to media framing, people tend to think first of asylum seekers or irregular migrants from poor non-western countries that denote cultural differences when hearing the word “immigrants” (Borrelli and Ruedin, 2024; Devos et al., 2010; Esses et al., 2013). While this gives our measure some face validity when it comes to the dynamics of political tolerance towards immigrants, future research should differentiate between immigrant groups for a more nuanced understanding of who is allowed to raise political critique in the eyes of citizens.

To better understand the contemporary rise in intolerance, our research underscores the need for a deeper engagement in the dialogue between the literature on immigration attitudes and the broader discussion about eroding democratic norms and support for civil liberties. Research on immigration attitudes has long highlighted the important role of socio-tropic and economic threats as powerful drivers of xenophobia or support for right-populist parties (Ceobanu and Escandell, 2010; Dunwoody and Plane, 2019; Erişen and Erdoğan, 2019; Green et al., 2016; Isaksen et al., 2016). Our research suggests that these grievances and threat considerations in combination with elevated levels of national or white identity, are also an important piece in the puzzle of eroding support for civil liberties and political tolerance. To learn more about the scope conditions of this argument, the theoretical reasoning and empirical tests presented in this study should be developed further.

Future research could extend beyond the immigrant treatment used in this study, to address the intersecting nature of markers of difference. Does it make a difference, if government critique is voiced by a woman with an immigrant background rather than a man? Or by a person of color with an immigrant background as opposed to a white immigrant? What about socio-demographic status? Is a cleaner viewed as equally entitled to voice critique as a lawyer with an immigrant background? Follow-up studies along these lines will eventually show whether a person’s immigrant background is the primary trigger of political intolerance for those who adhere to a threatened, identitarian view of the political community, as we would expect, or if it is merely one of many other markers reflecting a marginalized position in society that triggers political intolerance.

## Data Availability

Data and replication materials are accessible here: https://osf.io/r7tz5/.

## Footnotes

^1^https://ygo-assets-websites-editorial-emea.yougov.net/documents/Reactions_to_Harris_Trump_Debate_poll_results.pdf#page=6 [last accessed: 22 Oct. 2024].

^2^https://ewscripps.brightspotcdn.com/76/25/c50efe0b45bf80f50cbbb6ed38a7/scripps-news-ipsos-poll.pdf [last accessed: 22 Oct. 2024].

^3^All experiments received IRB approval. For pre-registration, see Appendix A.

^4^Based on the two countries’ traditional approaches to immigrant inclusion–a multicultural jus soli citizenship regime in the US vs. assimilationist jus sanguinis in Switzerland (Manatschal and Bernauer, 2016)–one might at first sight expect that immigrants are more easily included in the demos in the US, and that they would therefore be more readily seen as legitimate critics of the government, than in Switzerland. However, an easier access to citizenship does not make immigrants automatically part of the ethnoracial ingroup. Our argument focuses on these competing social identities around ascriptive understandings of membership in the demos, not on legalistic national group definitions.

^5^The US and Swiss surveys were fielded by Lucid, a non-probability online panel provider that works with partner firms to produce samples that are close to representative of American and Swiss adults (see Coppock and McClellan (2019) on external validity of findings based on Lucid samples). In Turkey, the survey was conducted online by Istanbul Economy Research, a Turkish-based research company, which maintains a research panel that is close to representative of the Turkish population. For all studies, we excluded respondents who complete the survey in less than 33% of the median response time.

^6^The phrasing “people critical of government” has been used in international studies of youth attitudes towards democracy, such as the IEA Civic Education Study, including studies conducted in Turkey. See Doğanay (2010) and Torney-Purta (2004). Our research design is meant to be conservative. We expect that when prompted with “people” most respondents think of people like themselves, and therefore citizens who belong to the native ethnic majority group. For example, in this scenario, a Trump supporter may think of other people like her who criticized the government. However, it could also be the case that the phrasing “people critical of government” may conjure up a disliked group, so, for example, our Trump supporter may think of supporters of Black Lives Matter or immigrant rights. Therefore, this phrasing leads to a conservative estimate because of the ambiguity inherent in the term “people.”

^7^We understand the restriction on voting (“being prevented from voting in elections”) and running for office (“being allowed to run for public office”) as exclusions from rights associated with naturalization. In all three countries, immigrants can be extended political rights through naturalization, and in some Swiss cantons and municipalities and US municipalities, even via non-citizen enfranchisement.

^8^The rating of each item was normalized between 0 and 1. In a second step, the index was created as an additive index capturing the average rating across the four tolerance items, without item specific weights.

^9^Our pre-registration included additional hypotheses that are not directly relevant to this paper. Specifically, we specified hypotheses with anti-immigrant attitudes and racial resentment as the key moderators. We have included these results in Appendix Tables I4, I5, and I6.

^10^All our data and stata codes are available here: https://osf.io/r7tz5/.

^11^https://theconversation.com/what-do-the-turkish-local-elections-mean-for-the-countrys-4-million-refugees-227478; https://www.aljazeera.com/news/2023/6/8/future-unknown-but-syrians-in-turkey-relieved-after-erdogan-win [last accessed: 23 Oct. 2024].
