# Supplementary material for: Are immigrants allowed to criticize the government? Ingroup identity, economic threat, and majority group support for immigrant civil liberties in the US, Switzerland, and Turkey
Source: Front Sociol. 2025 May 23;10:1520889. doi: 10.3389/fsoc.2025.1520889 (PMC12141267; doi:10.3389/fsoc.2025.1520889)
Supplement: Supplementary file 1 [file Data_Sheet_1.pdf]

Appendix

Supplementary material for: Gandenberger MK, Buyuker BE, Manatschal A and Filindra A (2025) Are immigrants allowed to criticize the government? Ingroup identity, economic threat, and majority group support for immigrant civil liberties in the US, Switzerland, and Turkey. Front. Sociol. doi: 10.3389/fsoc.2025.1520889

A. Pre-registration ***3***

B. Study set up ***5***

Item wording **5**

C. Summary statistics ***8***

Table C1. Summary statistics – Study 1 (US) **8**

Table C2. Summary statistics – Study 2 (CH) **8**

Table C3. Summary statistics – Study 3 (TUR) **9**

D. Histograms and descriptives dependent variables ***10***

E. Histograms key moderators ***13***

F. Correlation analyses key moderators and independent variables ***15***

Table F1. Correlation analyses – Study 1 (US) **15**

Table F2. Correlation analyses – Study 2 (CH) **16**

Table F3. Correlation analyses – Study 3 (TUR) **16**

G. Balance tables ***17***

Table G1. Balance table, mean by experiment group – Study 1 (US) **17**

Table G2. Balance table, mean by experiment group – Study 2 (CH) **17**

Table G3. Balance table, mean by experiment group – Study 3 (TUR) **18**

H. Main effects models ***19***

Table H1. Robust Regression Results, Main Effects - Study 1 (US) **19**

Table H2. Robust Regression Results, Main Effects - Study 2 (CH) **20**

Table H3. Robust Regression Results, Main Effects - Study 3 (TUR) **21**

I. Moderation models results ***22***

Table I1. Robust Regression Results, Moderation analyses: white identity, economic threat and white victimhood - Study 1 (US) **22**

Table I2. Robust Regression Results, Moderation analyses: national identity and economic threat - Study 2 (CH) **24**

Table I3. Robust Regression Results, Moderation analyses: national identity and economic threat - Study 3 (TUR) **26**

Additional tests on outgroup perceptions as moderators (racial resentment and xenophobia) . **28**

Table I4. Robust Regression Results, Main Effects and Interaction Models with Racial Resentment and Anti-Immigrant Attitudes - Study 1 (US) 29

Table I5. Robust Regression Results, Main Effects and Interaction Models with Anti-Immigrant Attitudes - Study 2 (CH) 30
Table I6. Robust Regression Results Main Effects and Interaction Models with Anti-Immigrant Attitudes - Study 3 (TUR) 31

J. Robustness checks 32

Table J1. Robust Regression Results, White identity: Interactions with Authoritarianism and Conservative Ideology - Study 1 (US) 32

Table J2. Robust Regression Results, White victimhood: Interactions with Authoritarianism and Conservative Ideology - Study 1 (US) 34

Table J3. Robust Regression Results, Including Interactions with Authoritarianism and Conservative Ideology - Study 2 (CH) 36

Table J4. Robust Regression Results, Including Interactions with Authoritarianism and Conservative Ideology - Study 3 (TUR) 38

A. Pre-registration

AS PREDICTED

Wharton CREDIBILITY LAB
UNIVERSITY of PENNSYLVANIA

CONFIDENTIAL – FOR PEER-REVIEW ONLY

NCCR-Political Tolerance (#56285)

Created: 01/20/2021 08:13 PM (PT)

This is an anonymized copy (without author names) of the pre-registration. It was created by the author(s) to use during peer-review.
A non-anonymized version (containing author names) should be made available by the authors when the work it supports is made public.

---

1) Have any data been collected for this study already?
No, no data have been collected for this study yet.

2) What's the main question being asked or hypothesis being tested in this study?
Scholars of political tolerance have shown that even though the public supports the principle of civil liberties, this support declines markedly when it comes to applying the rule to "disliked" groups (e.g., Sullivan et al. 1982, Gibson 2006, Peffley et al. 2015). Much of this work has focused on civil liberties of ideologically different or extreme groups such as racists, communists, and atheists but not neutral groups.Furthermore, these studies have not explored the effects of outgroup priming on political intolerance.

We seek to test whether outgroup priming with neutral primes can lead to more political intolerance, specifically among those who score high on outgroup bias and ingroup favorability.

We expect a main effect of our outgroup prime on political intolerance and a conditional effect based on one's prior levels of out-group bias and ingroup favorability. Specifically;

H1: Those who are exposed to the outgroup prime will become more politically intolerant than when in the control condition.

H2: Those who score high on racial resentment will be more likely to have higher levels of political intolerance when exposed to the outgroup prime than when in the control condition.

H3: Those who score high on xenophobia will be more likely to have higher levels of political intolerance when exposed to the outgroup prime than when in the control condition.

H4: Those who score high on in-group identity will be more likely to have higher levels of political intolerance when exposed to the outgroup prime than when in the control condition.

3) Describe the key dependent variable(s) specifying how they will be measured.
Political intolerance will be measured based on the standard battery (Gibson 1992, GSS, LAPOP).
- Be prevented from voting in elections
- Be allowed to organize protest marches and demonstrations
- Be allowed to run for public office
- Be allowed to teach in a college or university

4) How many and which conditions will participants be assigned to?
In this framing experiment, (1) the group that say critical things about government is manipulated.

Subjects are randomly assigned to one of two conditions:

(1) There are people who only say very critical things about the American/Swiss/Turkish form of government, not just parliament or the political parties, but the system of government. How strongly do you approve or disapprove that such people be allowed to do each of the following?

(2) There are immigrants who only say very critical things about the American/Swiss/Turkish form of government, not just parliament or the political parties, but the system of government. How strongly do you approve or disapprove that such people be allowed to do each of the following?

5) Specify exactly which analyses you will conduct to examine the main question/hypothesis.
We will use factor analyses to determine which of the four items to include in the overall political intolerance index?
We will use OLS regression modeling to specify models with interactions between the treatment variable and racial resentment and xenophobia.
For robustness checks, we will use interactions with alternate measures of out-group attitudes such as the social distance battery also included in the survey

6) Describe exactly how outliers will be defined and handled, and your precise rule(s) for excluding observations.
We will calculate the overall time that individuals used to complete the survey. Respondents who are within two standard deviations will be kept as valid.
The survey also includes attention checks. Respondents who fail all attention checks will be excluded.
The survey includes two open ended questions. Recent research by the Pew Center suggests that low quality respondents that may come from outside the country tend to write up generic responses in the open-ended section, such as "great product". We will review the open-ends and eliminate such cases.

7) How many observations will be collected or what will determine sample size? No need to justify decision, but be precise about exactly how the number will be determined.
The total sample size will be 704 per country. For the purposes of this project, the sample will be members of racial & ethnic majority groups. The sample size is determined based on power analyses to support models with interactions.

8) Anything else you would like to pre-register? (e.g., secondary analyses, variables collected for exploratory purposes, unusual analyses planned?)

Link to the pre-registration: https://aspredicted.org/TYF_94C

B. Study set up

Item wording

Moderator: White Identity (US only)

- How important to your identity is being of white European heritage? Extremely important; Very important; Moderately important; Slightly important; Not at all important
- When talking about people of white European heritage, how often do you say "we" instead of they? Always; Often; Sometimes; Rarely; Never
- How proud are you being white? Extremely proud; Very proud; Moderately proud; Slightly proud; Not proud at all

Moderator: National Identity (CH, TUR)

- How important is it for your identity to be [Swiss/Turkish]? Extremely important; Very important; Moderately important; Somewhat important; Not at all important
- When you talk about people of [Swiss/Turkish] origin, how often do you say 'we' instead of 'they'? Always; Often; Sometimes; Rarely; Never
- How proud are you being [Swiss/Turkish]? Extremely proud; Very proud; Moderately proud; Slightly proud; Not proud at all

Moderator: White Victimhood (US only)

Please indicate if you agree strongly, agree somewhat, disagree somewhat, or disagree strongly with each of the following statements.

- Talking about racial issues causes unnecessary tension
- Racial and ethnic minorities do not have the same opportunities as white people in the U.S
- White people in the U.S. are discriminated against because of the color of their skin

Moderator: Economic deprivation

How financially well off do you consider yourself to be compared to people like you 30 years ago? Much better; Somewhat better; About the same; Somewhat worse; Much worse

Moderator: Anti-immigrant attitudes

There are different opinions about immigrants from other countries living in [COUNTRY]. Please indicate if you agree strongly, agree somewhat, disagree somewhat, or disagree strongly with each of the following statements.

- Immigrants cause crime rates to increase
- Immigrants are generally good for [COUNTRY NATIONALITY] economy
- Immigrants take jobs away from [COUNTRY NATIONALITY] people
- [COUNTRY's] culture is generally undermined by immigrants
- Legal immigrants who are not citizens should have the same rights as [COUNTRY's] citizens
- [COUNTRY] should take stronger measures to exclude illegal immigration.
- Do you think the number of immigrants to [COUNTRY] nowadays should be increased a lot, increased a little, remain the same as it is, reduced a little, reduced a lot?

Moderator: Racial Resentment

For each of the following statements, please indicate if you think the statement is very true, somewhat true, slightly true, or not at all true.

- Irish, Italians, Jewish and many other minorities overcame prejudice and worked their way up. Blacks should do the same without any special favors
- Generations of slavery and discrimination have created conditions that make it difficult for blacks to work their way out of the lower class
- Over the past few years, blacks have gotten less than they deserve
- It's really a matter of some people not trying hard enough; if blacks would only try harder, they could be just as well off as Whites.

Authoritarianism

- Which one do you think is more important for a child to have? Independence; Respect for elders
- Which one do you think is more important for a child to have? Curiosity; Good manners
- Which one do you think is more important for a child to have? Being considerate; Well behaved
- Which one do you think is more important for a child to have? Obedience; Self-reliance

Ideology

- Generally speaking, do you consider yourself a liberal, moderate, or conservative? (US) Very liberal, somewhat liberal, moderate, somewhat conservative, very conservative
- In political matters, people talk often about "the left" and "the right". On a scale from 1 (left) to 10 (right), how would you place your views in this scale? (Switzerland)

Party ID

- Do you consider yourself a Strong Democrat, Weak Democrat, Leaning Democrat, Independent, Leaning Republican, Weak Republican, Strong Republican? (US)
- Which party did you vote for in the 2019 federal elections? (Switzerland) Freisinnig-Demokratische Partei; Christlichdemokratische Volkspartei; Sozialdemokratische Partei; Schweizerische Volkspartei; Evangelische Volkspartei; Christlich-soziale Partei; Partei der Arbeit der Schweiz; Grünliberale Partei; Mouvement citoyens genevois; Grüne Partei; Schweizer Demokraten; Eidgenössisch-Demokratische Union; Lega dei Ticinesi; Bürgerlich-Demokratische Partei; Alternative Linke; Did not vote
    - Coded as Left(0)/Center(0.5)/Far Right(1) following Bornschier et al. (2021) and Strijbis (2011)
        - Left: Grüne Partei, Sozialdemokratische Partei, Alternative Linke, Partei der Arbeit der Schweiz
        - Center: Christlichdemokratische Volkspartei, Freisinnig-Demokratische Partei, Grünliberale Partei, Bürgerlich-Demokratische Partei, Evangelische Volkspartei

        - Far Right: Schweizerische Volkspartei, Eidgenössisch-Demokratische Union, Schweizer Demokraten, Lega dei Ticinesi, Mouvement citoyens genevois
- Which party did you vote for in the most recent general elections? (Turkey) AKP (Adalet ve Kalkinma Partisi); CHP (Cumhuriyey Halk Partisi); MHP (Milliyetci Hareket Partisi); Iyi Parti; HDP (Halklarin Demokratik Partisi); SP (Saadet Partisi); None of them
    - Coded as Left(0)/Center(0.5)/Far Right(1) following Kalaycioglu (2015) and Moral (2021)
        - Left: Halklarin Demokratik Partisi
        - Center: Cumhuriyey Halk Partisi, Iyi Parti
        - Right: Adalet ve Kalkinma Partisi, Milliyetci Hareket Partisi, Saadet Partisi

<u>Gender</u>

Are you... Male/Female?

<u>Education</u>

- What is the last grade of school you completed?

  Less than high school; High school; Some college; College degree; Some graduate education Post graduate degree

<u>Age</u>

- What is your age?

<u>Income</u>

- In which group does your total household income fall?

C. Summary statistics

Table C1. Summary statistics – Study 1 (US)

Variable	Obs	Mean	Std. dev.	Min	Max
Intolerance index	6,762	0.400436	0.26238	0	1
White ID	6,762	0.519681	0.283813	0	1
Economic Threat	6,762	0.381544	0.410107	0	1
White Victimhood	6,762	0.520096	0.238937	0	1
Racial resentment	6,762	0.535875	0.289609	0	1
Anti-immigrant attitudes	6,762	0.499106	0.218118	0	1
Age 18-29	6,762	0.108104	0.310535	0	1
Age 30-44	6,762	0.348861	0.476645	0	1
Age 45-64	6,762	0.298285	0.457539	0	1
Age 65+	6,762	0.24475	0.429971	0	1
College degree	6,762	0.560781	0.496329	0	1
Female	6,762	0.520704	0.499608	0	1
income	6,762	0.453712	0.49789	0	1
Partisanship	6,762	0.474192	0.366251	0	1
Conservative ideology	6,762	0.5332	0.312734	0	1
Authoritarianism	6,762	0.507912	0.318581	0	1

Table C2. Summary statistics – Study 2 (CH)

Variable	Obs	Mean	Std. dev.	Min	Max
Intolerance Index	2,392	0.40632	0.180408	0	1
National ID	2,392	0.719029	0.19746	0	1
Economic Threat	2,392	0.376463	0.427879	0	1
Anti-immigrant attitudes	2,392	0.526208	0.157617	0	1
Age 18-29	2,392	0.175585	0.380547	0	1
Age 30-44	2,392	0.239967	0.427152	0	1
Age 45-64	2,392	0.431856	0.495438	0	1
Age 65+	2,392	0.152592	0.359669	0	1
University degree	2,392	0.570652	0.495087	0	1
Female	2,392	0.480769	0.499735	0	1
Income	2,392	0.768395	0.421946	0	1
Partisanship	2,069	0.458676	0.355406	0	1
Authoritarianism	2,392	0.468646	0.248919	0	1
Conservative Ideology	2,392	0.61505	0.225533	0	1

Table C3. Summary statistics – Study 3 (TUR)

Variable	Obs	Mean	Std. dev.	Min	Max
Intolerance index	2,826	0.497818	0.225545	0	1
National ID	2,826	0.837993	0.20693	0	1
Economic Threat	2,826	0.4908	0.465922	0	1
Anti-immigrant attitudes*	2,826	0.71298	0.160586	0.166667	1
Age 18-29	2,826	0.313164	0.463862	0	1
Age 30-44	2,826	0.408705	0.491682	0	1
Age 45-64	2,826	0.214791	0.41075	0	1
Age 65+	2,826	0.06334	0.243617	0	1
University degree	2,826	0.486553	0.499908	0	1
Female	2,826	0.487261	0.499926	0	1
Income	2,826	0.094834	0.293037	0	1
Partisanship	2,226	0.752471	0.271584	0	1
Authoritarianism	2,826	0.441879	0.293448	0	1
Conservative ideology	2,826	0.508807	0.299479	0	1

*

D. Histograms and descriptives dependent variables

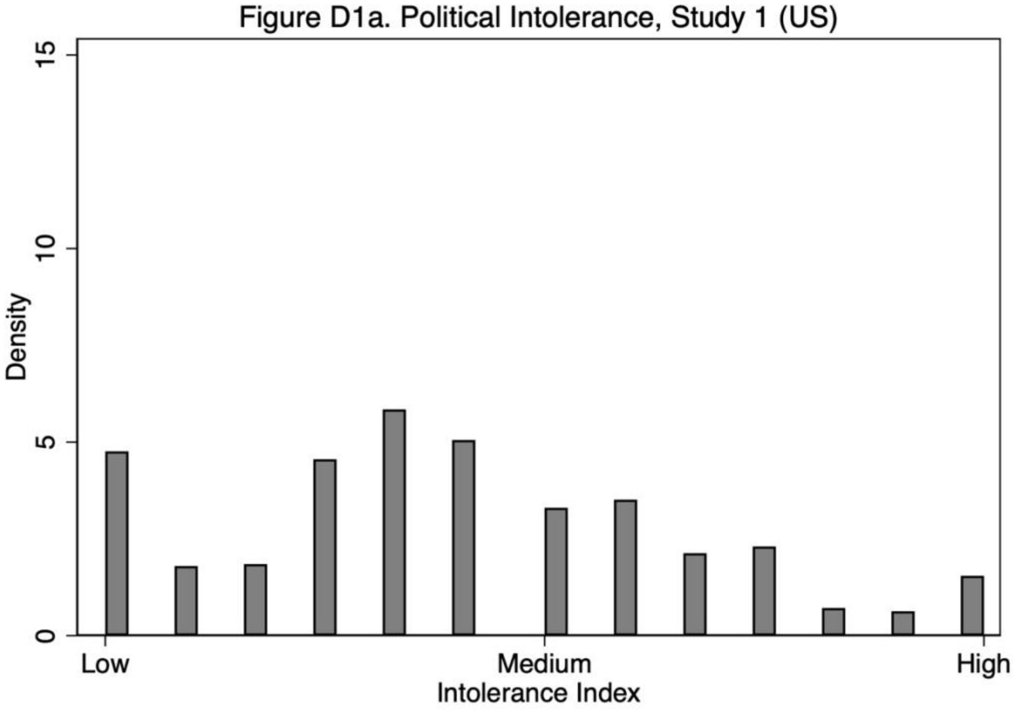

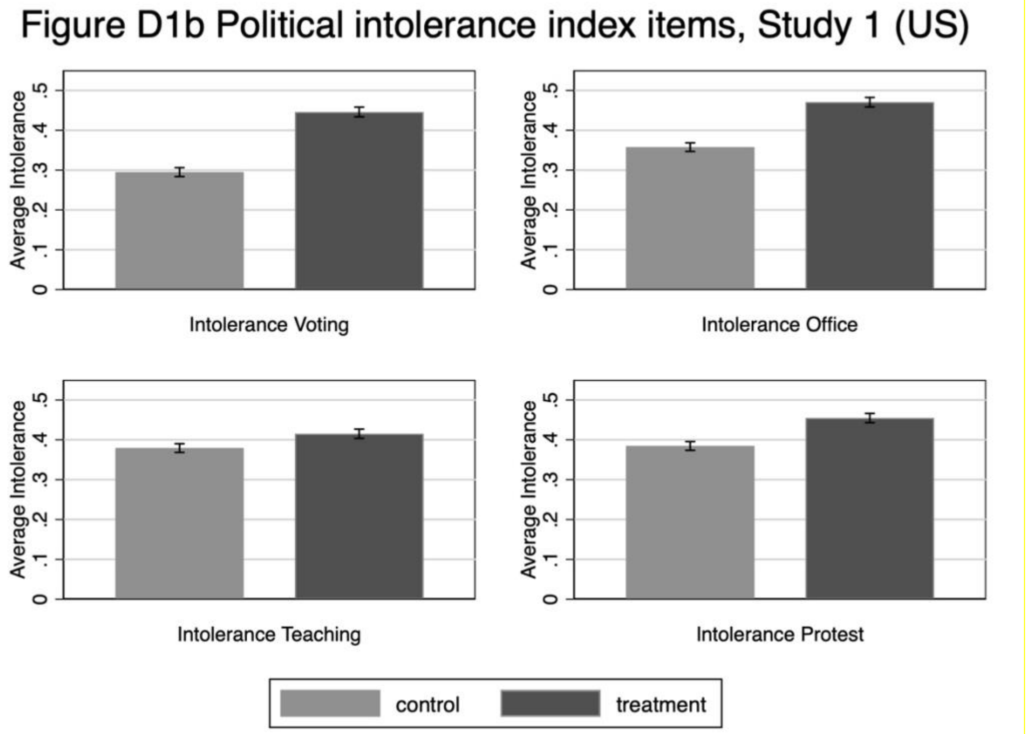

Figure D2a. Political Intolerance, Study 2 (CH)

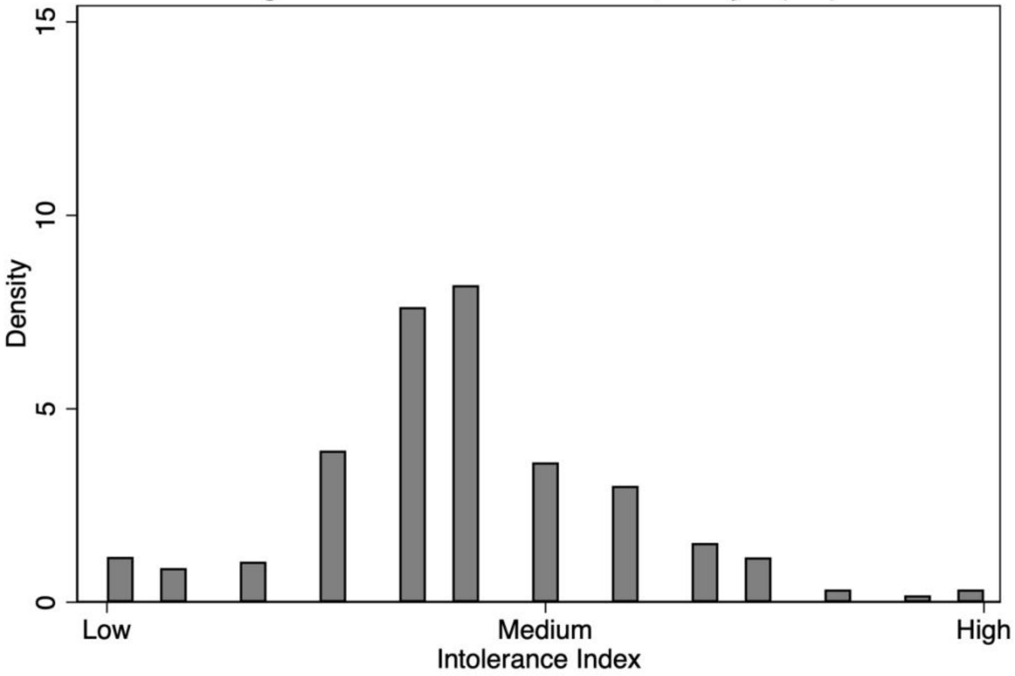

Figure D2b. Political intolerance index items, Study 2 (CH)

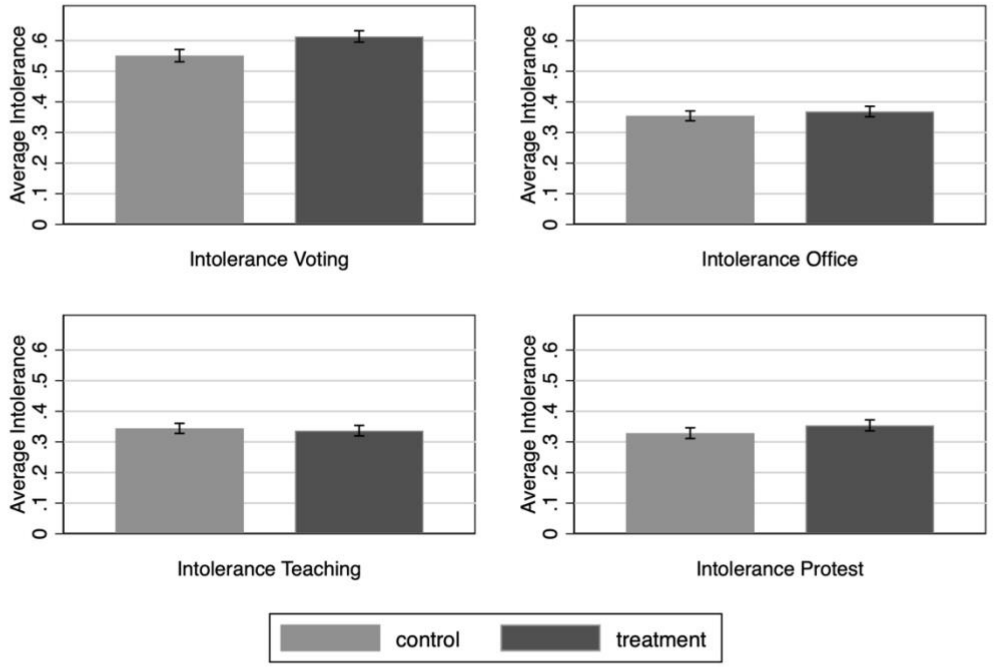

Figure D3a. Political Intolerance, Study 3 (TUR)

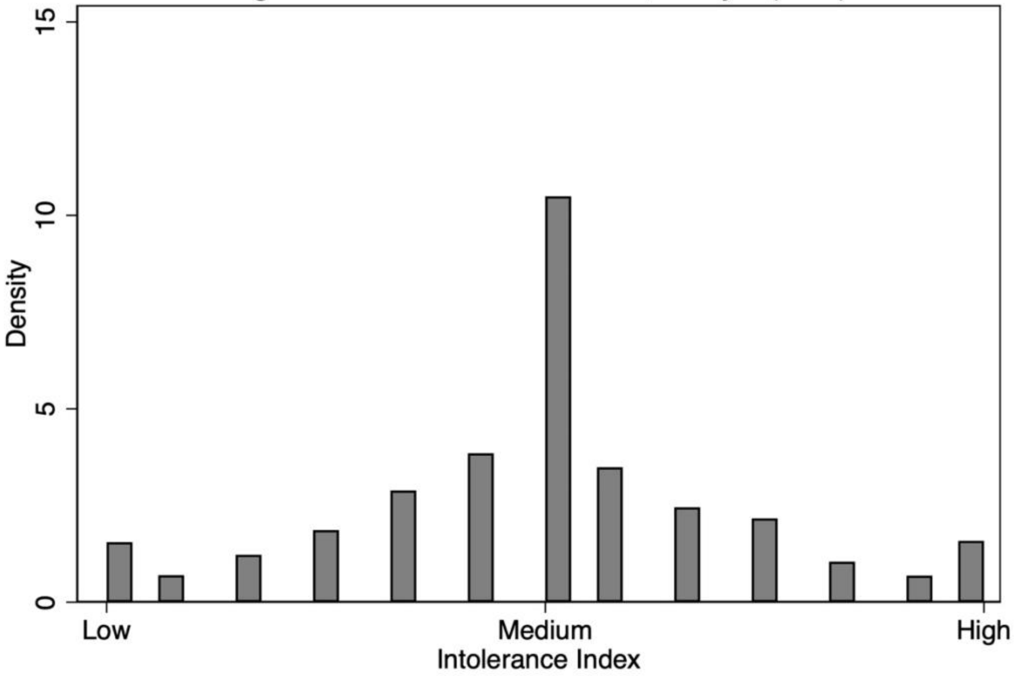

Figure D3b. Political intolerance index items, Study 3 (TUR)

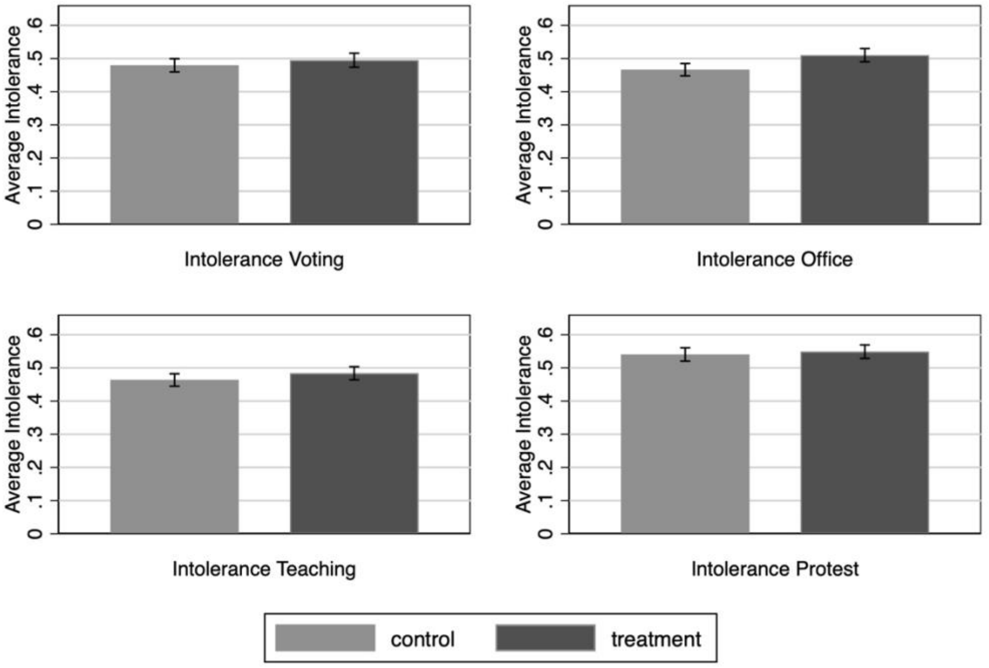

E. Histograms key moderators

Study 1 (US)

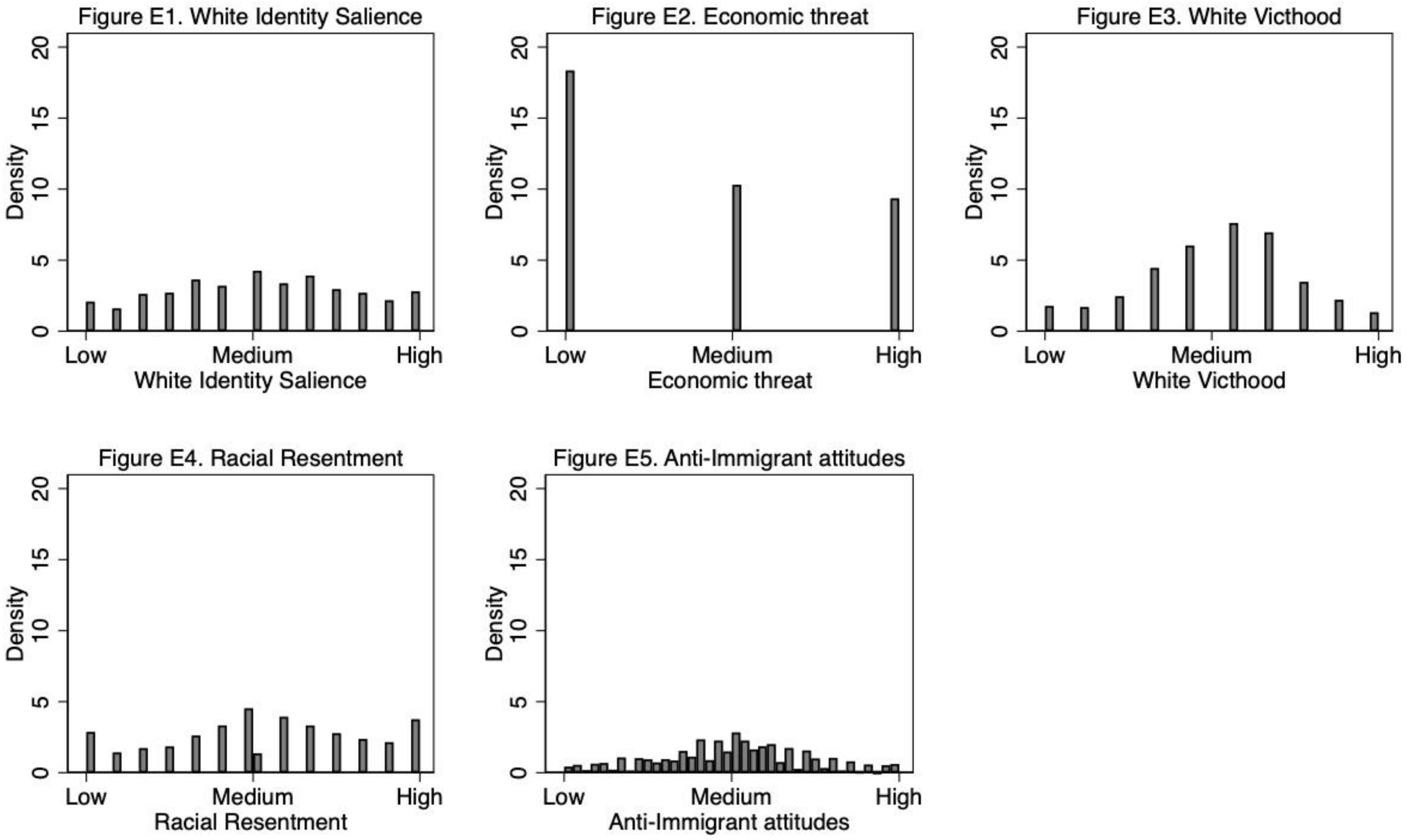

Study 2 (CH)

Figure E6. National Identity Salience

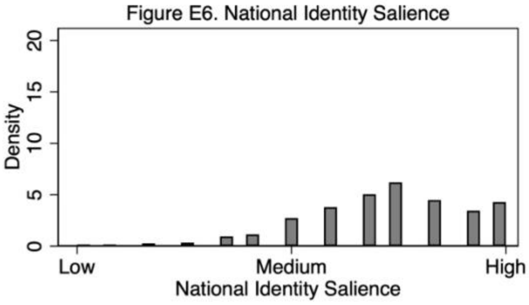

Figure E7. Economic threat

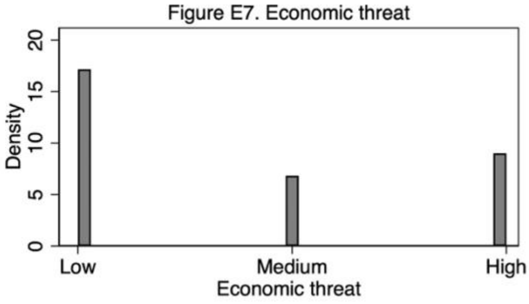

Figure E8. Anti-Immigrant attitudes

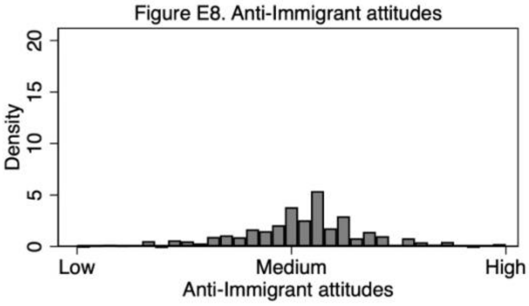

Study 3 (TUR)

Figure E9. National Identity Salience

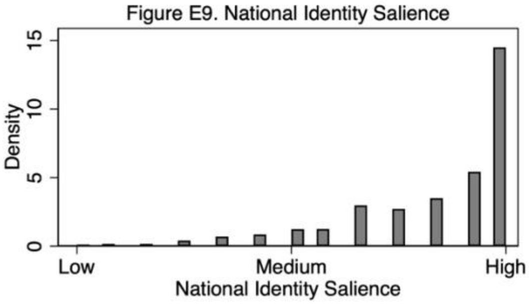

Figure E10. Economic deprivation

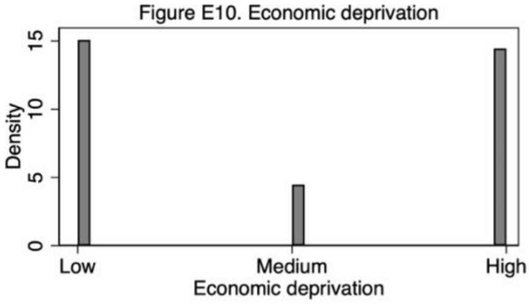

Figure E11. Anti-Immigrant attitudes

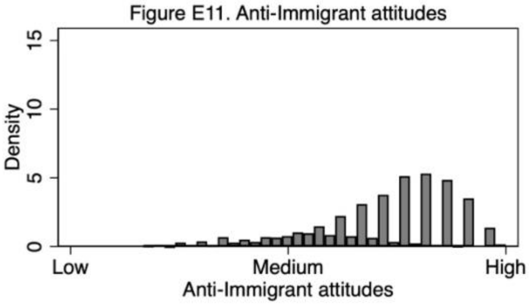

F. Correlation analyses key moderators and independent variables

Table F1. Correlation analyses – Study 1 (US)

	White ID	Economic Threat	Anti-immigrant attitudes	Racial resentment	White Victimhood	Partisanship	Conservative ideology
White ID	1						
Economic Threat	-0.2593	1					
Anti-immigrant attitudes	0.3204	-0.0303	1				
Racial resentment	0.2482	-0.0326	0.6054	1			
White Victimhood	0.2771	-0.0383	0.5888	0.6778	1		
Partisanship	0.0121	0.0391	0.4492	0.4615	0.3985	1	
Conservative ideology	0.2912	-0.1456	0.452	0.457	0.4653	0.4685	1

Table F2. Correlation analyses – Study 2 (CH)

	National ID	Economic Threat	Anti-immigrant attitudes	Partisanship	Conservative Ideology
National ID	1				
Economic Threat	-0.089	1			
Anti-immigrant attitudes	0.3552	0.0012	1		
Partisanship	0.142	-0.0028	0.2359	1	
Conservative Ideology	0.3892	-0.1901	0.3887	0.1658	1

Table F3. Correlation analyses – Study 3 (TUR)

	National ID	Economic Threat	Anti-immigrant attitudes	Partisanship	Conservative ideology
National ID	1				
Economic Threat	-0.1082	1			
Anti-immigrant attitudes	0.0792	0.2043	1		
Partisanship	0.3261	-0.3065	-0.1091	1	
Conservative ideology	0.2436	-0.2516	-0.1339	0.5214	1

G. Balance tables

Table G1. Balance table, mean by experiment group – Study 1 (US)

	Experiment group	
	Control	Treatment
Intolerance index	0.354111	0.446652
White ID	0.514707	0.524643
Economic Threat	0.380219	0.382866
White Victimhood	0.522686	0.517512
Racial resentment	0.538077	0.533679
Anti-immigrant attitudes	0.501625	0.496592
Partisanship	0.472508	0.475872
Conservative ideology	0.528477	0.537912
Authoritarianism	0.511771	0.504062
Female	0.514362	0.527031
Age 18-29	0.106307	0.109897
Age 30-44	0.350903	0.346824
Age 45-64	0.296417	0.300148
Age 65+	0.246373	0.243132
Income	0.45573	0.451699
College degree	0.558484	0.563072
N	3,377	3,385

Table G2. Balance table, mean by experiment group – Study 2 (CH)

	Experiment group	
	Control	Treatment
Intolerance Index	0.394458	0.418201
National ID	0.719089	0.718968
Economic Threat	0.393484	0.359414
Anti-immigrant attitudes	0.522984	0.529438
Partisanship	0.459499	0.457849
Conservative Ideology	0.605932	0.624184
Authoritarianism	0.467627	0.469665
Female	0.472013	0.48954
Age 18-29	0.167084	0.1841
Age 30-44	0.244779	0.235146
Age 45-64	0.429407	0.43431
Age 65+	0.15873	0.146444
Income	0.772765	0.764017
University degree	0.575606	0.56569
N	1,197	1,195

Table G3. Balance table, mean by experiment group – Study 3 (TUR)

	Experiment group	
	Control	Treatment
Intolerance index	0.487416	0.509419
National ID	0.83585	0.840382
Economic Threat	0.474161	0.509356
Anti-immigrant attitudes	0.706456	0.720256
Partisanship	0.764731	0.738863
Conservative ideology	0.514243	0.502745
Authoritarianism	0.445973	0.437313
Female	0.492617	0.481287
Age 18-29	0.330873	0.293413
Age 30-44	0.402685	0.415419
Age 45-64	0.202013	0.229042
Age 65+	0.06443	0.062126
Income	0.112081	0.075599
University degree	0.469799	0.50524
N	1,490	1,336

H. Main effects models

Table H1. Robust Regression Results, Main Effects - Study 1 (US)

	Model 1	*Model 2*
	b/se	b/se
Treatment	0.093***	0.093***
	(0.01)	(0.01)
Age 30-44		0.072***
		(0.01)
Age 45-64		0.046***
		(0.01)
Age 65+		0.045***
		(0.01)
College Degree		-0.044***
		(0.01)
Female		0.048***
		(0.01)
Income		-0.025***
		(0.01)
Economic Threat (3 levels)		-0.005
		(0.01)
Partisanship		0.071***
		(0.01)
Conservative Ideology		0.096***
		(0.01)
Authoritarianism		0.181***
		(0.01)
Constant	0.346***	0.135***
	(0.00)	(0.01)
N	6762	6762
Adj.R-squared	0.028	0.156
F	195.132	114.407

Notes: Non-Hispanic White Americans only. Robust standard errors in parentheses. All predictors are coded on a 0 to 1 scale, so all coefficients can be thought as maximum effects. ***p<0.01; ** p<0.05; * p<0.1 (two-tailed)

Table H2. Robust Regression Results, Main Effects - Study 2 (CH)

	Model 1	*Model 2*
	b/se	b/se
Treatment	0.005	0.007
	(0.01)	(0.01)
Age 30-44		0.007
		(0.01)
Age 45-64		0.046***
		(0.01)
Age 65+		0.045***
		(0.01)
University Degree		-0.043***
		(0.01)
Female		-0.028***
		(0.01)
Income		0.012
		(0.01)
Economic Threat (3 levels)		0.009
		(0.01)
Partisanship		0.042***
		(0.01)
Conservative ideology		0.038**
		(0.02)
Authoritarianism		0.114***
		(0.01)
Constant	0.394***	0.290***
	(0.00)	(0.02)
N	2392	2069
Adj.R-squared	-0.000	0.095
F	0.576	20.710

Notes: German-speaking Swiss only. Robust standard errors in parentheses. All predictors are coded on a 0 to 1 scale, so all coefficients can be thought as maximum effects. ***p<0.01; ** p<0.05; * p<0.1 (two-tailed)

Table H3. Robust Regression Results, Main Effects - Study 3 (TUR)

	Model 1	*Model 2*
	b/se	b/se
Treatment	0.020**	0.029***
	(0.01)	(0.01)
Age 30-44		0.013
		(0.01)
Age 45-64		-0.032**
		(0.01)
Age 65+		0.012
		(0.02)
University Degree		-0.021**
		(0.01)
Female		0.019**
		(0.01)
Income		0.002
		(0.02)
Economic Threat (3 levels)		0.011
		(0.01)
Partisanship		0.068***
		(0.02)
Conservative Ideology		0.011
		(0.02)
Authoritarianism		0.028
		(0.02)
Constant	0.487***	0.406***
	(0.01)	(0.02)
N	2826	2226
Adj.R-squared	0.002	0.023
F	5.742	5.681

Notes: Turkish citizens only. Robust standard errors in parentheses. All predictors are coded ona 0 to 1 scale, so all coefficients can be thought as maximum effects. ***p<0.01; ** p<0.05; * p<0.1 (two-tailed)

I. Moderation models results

Table I1. Robust Regression Results, Moderation analyses: white identity, economic threat and white victimhood - Study 1 (US)

	White Identity		Economic threat		Interactions	Three-way interaction	Interaction Econ. threat (high)	White victimhood		Three-way interaction	Interaction Econ. threat (high)
	Model 1	*Model 2*	*Model 3*	*Model 4*	*Model 5*	*Model 6*	*Model 7*	*Model 8*	*Model 9*	Model 10	Model 11
	b/se	b/se	b/se	b/se	b/se	b/se	b/se	b/se	b/se	b/se	b/se
Treatment	0.092***	0.082***	0.093***	0.082***	0.061***	0.097***	0.053**	0.094***	-0.011	-0.002	-0.019
	(0.01)	(0.01)	(0.01)	(0.01)	(0.02)	(0.02)	(0.02)	(0.01)	(0.01)	(0.02)	(0.03)
White identity	0.129***	0.120***			0.113***	0.132***	0.029				
	(0.01)	(0.02)			(0.02)	(0.02)	(0.03)				
Age 30-44	0.059***	0.059***	0.072***	0.072***	0.059***	0.058***	0.050**	0.055***	0.055***	0.053***	0.033
	(0.01)	(0.01)	(0.01)	(0.01)	(0.01)	(0.01)	(0.02)	(0.01)	(0.01)	(0.01)	(0.02)
Age 45-64	0.045***	0.045***	0.046***	0.046***	0.045***	0.044***	0.053**	0.032***	0.032***	0.030***	0.036
	(0.01)	(0.01)	(0.01)	(0.01)	(0.01)	(0.01)	(0.02)	(0.01)	(0.01)	(0.01)	(0.02)
Age 65+	0.049***	0.049***	0.045***	0.045***	0.049***	0.048***	0.059**	0.042***	0.042***	0.040***	0.053**
	(0.01)	(0.01)	(0.01)	(0.01)	(0.01)	(0.01)	(0.02)	(0.01)	(0.01)	(0.01)	(0.02)
College Degree	-0.046***	-0.046***	-0.044***	-0.044***	-0.046***	-0.045***	-0.035**	-0.036***	-0.036***	-0.036***	-0.028**
	(0.01)	(0.01)	(0.01)	(0.01)	(0.01)	(0.01)	(0.01)	(0.01)	(0.01)	(0.01)	(0.01)
Female	0.051***	0.051***	0.048***	0.048***	0.051***	0.051***	0.072***	0.054***	0.054***	0.055***	0.078***
	(0.01)	(0.01)	(0.01)	(0.01)	(0.01)	(0.01)	(0.01)	(0.01)	(0.01)	(0.01)	(0.01)
Income Binary	-0.027***	-0.027***	-0.025***	-0.025***	-0.027***	-0.027***	-0.014	-0.024***	-0.024***	-0.024***	-0.008
	(0.01)	(0.01)	(0.01)	(0.01)	(0.01)	(0.01)	(0.02)	(0.01)	(0.01)	(0.01)	(0.02)
Economic Threat (3 levels)	0.012	0.012	-0.005	-0.021*	-0.007	0.019		-0.001	-0.001	-0.044*	
	(0.01)	(0.01)	(0.01)	(0.01)	(0.01)	(0.02)		(0.01)	(0.01)	(0.02)	
Partisanship	0.077***	0.077***	0.071***	0.071***	0.076***	0.075***	0.053*	0.043***	0.043***	0.043***	0.032

	(0.01)	(0.01)	(0.01)	(0.01)	(0.01)	(0.01)	(0.03)	(0.01)	(0.01)	(0.01)	(0.03)
Conservative ideology	0.082***	0.082***	0.096***	0.096***	0.082***	0.082***	0.136***	0.048***	0.050***	0.047***	0.063*
	(0.01)	(0.01)	(0.01)	(0.01)	(0.01)	(0.01)	(0.03)	(0.01)	(0.01)	(0.01)	(0.03)
Authoritarianism	0.155***	0.155***	0.181***	0.181***	0.155***	0.155***	0.237***	0.146***	0.146***	0.143***	0.209***
	(0.01)	(0.01)	(0.01)	(0.01)	(0.01)	(0.01)	(0.02)	(0.01)	(0.01)	(0.01)	(0.02)
**Treatment*White identity**		0.018			0.033	-0.029	**0.125*** **				
		(0.02)			(0.02)	(0.03)	**(0.05)**				
**Treatment*Economic Threat (3 levels)**				**0.032** **	**0.038** **	-0.047				-0.014	
				**(0.01)**	**(0.02)**	(0.03)				(0.03)	
Economic Threat (3 levels)*White identity						-0.053					
						(0.04)					
**Treatment*Economic Threat (3 levels)*White identity**						**0.171*** **					
						**(0.05)**					
White victimhood								0.236***	0.132***	0.113***	0.113***
								(0.01)	(0.02)	(0.03)	(0.04)
**Treatment*White victimhood**									**0.206*** **	**0.160*** **	**0.265*** **
									**(0.02)**	**(0.04)**	**(0.05)**
White victimhood*Economic Threat (3 levels)										0.046	
										(0.04)	
**Treatment*White victimhood*Economic Threat (3 levels)**										**0.111***	
										**(0.06)**	
Constant	0.084***	0.089***	0.135***	0.141***	0.099***	0.090***	0.050*	0.070***	0.122***	0.143***	0.066**
	(0.01)	(0.02)	(0.01)	(0.01)	(0.02)	(0.02)	(0.03)	(0.01)	(0.02)	(0.02)	(0.03)
N	6762	6762	6762	6762	6762	6762	1663	6762	6762	6762	1663
Adj.R-squared	0.170	0.171	0.156	0.156	0.171	0.173	0.235	0.188	0.199	0.202	0.268
F	116.777	107.908	114.407	105.465	100.906	89.417	43.632	131.640	129.884	108.033	51.662

Notes: Non-Hispanic White Americans only. Robust standard errors in parentheses. All predictors are coded on a 0 to 1 scale so all coefficients can be thought as maximum effects. ***p<0.01; ** p<0.05; * p<0.1 (two-tailed)

Table I2. Robust Regression Results, Moderation analyses: national identity and economic threat - Study 2 (CH)

	National identity		Economic threat		Interactions	Three-way interaction	Interaction Econ. threat (high)
	Model 1	*Model 2*	*Model 3*	*Model 4*	*Model 5*	*Model 6*	*Model 7*
	b/se	b/se	b/se	b/se	b/se	b/se	b/se
Treatment	0.007	-0.028	0.007	-0.002	-0.040	-0.049	0.004
	(0.01)	(0.03)	(0.01)	(0.01)	(0.03)	(0.04)	(0.06)
National identity	0.013	-0.012			-0.013	-0.034	0.030
	(0.02)	(0.03)			(0.03)	(0.04)	(0.05)
Age 30-44	0.006	0.006	0.007	0.006	0.005	0.005	-0.030
	(0.01)	(0.01)	(0.01)	(0.01)	(0.01)	(0.01)	(0.03)
Age 45-64	0.045***	0.045***	0.046***	0.045***	0.044***	0.044***	0.015
	(0.01)	(0.01)	(0.01)	(0.01)	(0.01)	(0.01)	(0.02)
Age 65+	0.044***	0.044***	0.045***	0.044***	0.043***	0.042***	0.018
	(0.01)	(0.01)	(0.01)	(0.01)	(0.01)	(0.01)	(0.03)
University Degree	-0.043***	-0.043***	-0.043***	-0.043***	-0.043***	-0.043***	-0.027*
	(0.01)	(0.01)	(0.01)	(0.01)	(0.01)	(0.01)	(0.02)
Female	-0.028***	-0.028***	-0.028***	-0.028***	-0.029***	-0.029***	-0.045***
	(0.01)	(0.01)	(0.01)	(0.01)	(0.01)	(0.01)	(0.02)
Income	0.011	0.012	0.012	0.011	0.011	0.012	0.012
	(0.01)	(0.01)	(0.01)	(0.01)	(0.01)	(0.01)	(0.02)
Economic Threat (3 levels)	0.009	0.009	0.009	-0.003	-0.004	-0.043	
	(0.01)	(0.01)	(0.01)	(0.01)	(0.01)	(0.04)	
Partisanship	0.042***	0.042***	0.042***	0.041***	0.041***	0.041***	0.035*
	(0.01)	(0.01)	(0.01)	(0.01)	(0.01)	(0.01)	(0.02)
Conservative ideology	0.034*	0.034*	0.038**	0.039**	0.035**	0.035**	0.052

	(0.02)	(0.02)	(0.02)	(0.02)	(0.02)	(0.02)	(0.04)
Authoritarianism	0.115***	0.114***	0.114***	0.114***	0.114***	0.114***	0.067**
	(0.01)	(0.01)	(0.01)	(0.01)	(0.01)	(0.01)	(0.03)
Treatment*National identity		0.047			0.051	0.062	0.042
		(0.04)			(0.04)	(0.05)	(0.08)
**Treatment*Economic Threat (3 levels)**				**0.027***	**0.028***	0.041	
				**(0.02)**	**(0.02)**	(0.07)	
Economic Threat (3 levels)*National identity						0.052	
						(0.06)	
Treatment*Economic Threat (3 levels)*National identity						-0.016	
						(0.09)	
Constant	0.284***	0.303***	0.290***	0.296***	0.310***	0.327***	0.309***
	(0.02)	(0.02)	(0.02)	(0.02)	(0.02)	(0.03)	(0.04)
N	2069	2069	2069	2069	2069	2069	539
Adj.R-squared	0.095	0.095	0.095	0.096	0.096	0.096	0.060
F	19.149	17.711	20.710	19.278	16.719	14.702	3.864

Notes: German-speaking Swiss only. Robust standard errors in parentheses. All predictors are coded on a 0 to 1 scale, so all coefficients can be thought as maximum effects. ***p<0.01; ** p<0.05; * p<0.1 (two-tailed)

Table I3. Robust Regression Results, Moderation analyses: national identity and economic threat - Study 3 (TUR)

	National identity		Economic threat		Interactions	Three-way interaction	Interaction Econ. Threat (high)
	Model 1	*Model 2*	*Model 3*	*Model 4*	*Model 5*	*Model 6*	*Model 7*
	b/se	b/se	b/se	b/se	b/se	b/se	b/se
Treatment	0.029***	-0.035	0.029***	-0.003	-0.086**	0.028	-0.071
	(0.01)	(0.04)	(0.01)	(0.01)	(0.04)	(0.07)	(0.07)
National Identity	0.130***	0.096***			0.087**	0.076	0.128**
	(0.03)	(0.03)			(0.03)	(0.05)	(0.06)
Age 30-44	0.008	0.008	0.013	0.013	0.007	0.007	0.017
	(0.01)	(0.01)	(0.01)	(0.01)	(0.01)	(0.01)	(0.03)
Age 45-64	-0.036**	-0.036**	-0.032**	-0.031**	-0.034**	-0.035**	-0.025
	(0.01)	(0.01)	(0.01)	(0.01)	(0.01)	(0.01)	(0.03)
Age 65+	0.006	0.005	0.012	0.012	0.005	0.004	0.015
	(0.02)	(0.02)	(0.02)	(0.02)	(0.02)	(0.02)	(0.04)
University Degree	-0.021**	-0.020**	-0.021**	-0.024**	-0.022**	-0.022**	-0.021
	(0.01)	(0.01)	(0.01)	(0.01)	(0.01)	(0.01)	(0.02)
Female	0.012	0.012	0.019**	0.016	0.008	0.007	-0.010
	(0.01)	(0.01)	(0.01)	(0.01)	(0.01)	(0.01)	(0.02)
Income	0.003	0.003	0.002	0.002	0.003	0.002	-0.032
	(0.02)	(0.02)	(0.02)	(0.02)	(0.02)	(0.02)	(0.03)
Economic Threat (3 levels)	0.012	0.011	0.011	-0.023	-0.025*	-0.045	
	(0.01)	(0.01)	(0.01)	(0.01)	(0.01)	(0.06)	
Partisanship	0.047**	0.046**	0.068***	0.067***	0.044*	0.043*	0.004
	(0.02)	(0.02)	(0.02)	(0.02)	(0.02)	(0.02)	(0.04)
Conservative Ideology	0.003	0.004	0.011	0.014	0.008	0.009	0.052
	(0.02)	(0.02)	(0.02)	(0.02)	(0.02)	(0.02)	(0.03)

Authoritarianism	0.012	0.011	0.028	0.027	0.010	0.008	0.032
	(0.02)	(0.02)	(0.02)	(0.02)	(0.02)	(0.02)	(0.04)
Treatment*National Identity		0.075			0.096**	-0.035	0.183**
		(0.05)			(0.05)	(0.07)	(0.08)
**Treatment*Economic Threat (3 levels)**				**0.073*** **	**0.077*** **	-0.133	
				**(0.02)**	**(0.02)**	(0.09)	
Economic Threat (3 levels)*National Identity						0.024	
						(0.07)	
**Treatment*Economic Threat (3 levels)*National Identity**						**0.245** **	
						**(0.10)**	
Constant	0.330***	0.360***	0.406***	0.423***	0.386***	0.398***	0.329***
	(0.03)	(0.03)	(0.02)	(0.02)	(0.03)	(0.05)	(0.06)
N	2226.0000	2226.0000	2226.0000	2226.0000	2226.0000	2226.0000	943.0000
Adj.R-squared	0.033	0.034	0.023	0.028	0.040	0.046	0.067
F	7.366	7.007	5.681	6.289	7.542	7.688	6.637

Notes: Turkish citizens only. Robust standard errors in parentheses. All predictors are coded on a 0 to 1 scale, so all coefficients can be thought as maximum effects. ***p<0.01; ** p<0.05; * p<0.1 (two-tailed)

Additional tests on outgroup perceptions as moderators (racial resentment and xenophobia)

We capture outgroup perceptions in all three countries as anti-immigrant attitudes. We measure these by constructing an additive index based on seven statements of attitudes towards immigrants that include both positively and negatively-valenced items (αUS=0.80; α_{CH}=0.72; α_{TR}=0.57). Additionally, in the US, we measure racial resentment relying by creating an index based on four items (αUS=0.76). To summarise: In all three countries those with higher anti-immigrant attitudes are more intolerant towards critique voiced by immigrants (TablesI4-I6). Additionally, in the US, those scoring high on racial resentment are significantly more intolerant towards immigrant critics than those scoring lower (see Table I4).

Table I4. Robust Regression Results, Main Effects and Interaction Models with Racial Resentment and Anti-Immigrant Attitudes - Study 1 (US)

	Racial resentment		Anti-immigrant attitudes	
	Model 1	*Model 2*	*Model 3*	*Model 4*
	b/se	b/se	b/se	b/se
Treatment	0.094***	-0.025**	0.096***	-0.065***
	(0.01)	(0.01)	(0.01)	(0.01)
Racial resentment	0.221***	0.108***		
	(0.01)	(0.02)		
Age 30-44	0.048***	0.046***	0.041***	0.039***
	(0.01)	(0.01)	(0.01)	(0.01)
Age 45-64	0.015	0.014	0.021**	0.019*
	(0.01)	(0.01)	(0.01)	(0.01)
Age 65+	0.022*	0.020*	0.032***	0.029***
	(0.01)	(0.01)	(0.01)	(0.01)
College Degree	-0.034***	-0.032***	-0.029***	-0.028***
	(0.01)	(0.01)	(0.01)	(0.01)
Female	0.054***	0.054***	0.052***	0.052***
	(0.01)	(0.01)	(0.01)	(0.01)
Income Binary	-0.025***	-0.026***	-0.024***	-0.024***
	(0.01)	(0.01)	(0.01)	(0.01)
Economic Threat (3 levels)	-0.001	-0.001	0.001	0.001
	(0.01)	(0.01)	(0.01)	(0.01)
Partisanship	0.030***	0.031***	0.004	0.004
	(0.01)	(0.01)	(0.01)	(0.01)
Conservative ideology	0.045***	0.047***	0.021*	0.022*
	(0.01)	(0.01)	(0.01)	(0.01)
Authoritarianism	0.142***	0.141***	0.107***	0.107***
	(0.01)	(0.01)	(0.01)	(0.01)
**Treatment*Racial resentment**		**0.229*** **		
		**(0.02)**		
Anti-immigrant Attitudes			0.469***	0.303***
			(0.02)	(0.02)
**Treatment*Anti-immigrant Attitudes**				**0.328*** **
				**(0.03)**
Constant	0.095***	0.155***	0.019	0.101***
	(0.01)	(0.01)	(0.01)	(0.01)
N	6762	6762	6762	6762
Adj.R-squared	0.194	0.212	0.256	0.275
F	136.265	141.208	194.747	198.014

Notes: Non-Hispanic White Americans only. Robust standard errors in parentheses. All predictors are coded on a 0 to 1 scale so all coefficients can be thought as maximum effects. ***p<0.01; ** p<0.05; * p<0.1 (two-tailed)

Table I5. Robust Regression Results, Main Effects and Interaction Models with Anti-Immigrant Attitudes - Study 2 (CH)

	Model 1	*Model 2*
	b/se	b/se
Treatment	0.008	-0.071***
	(0.01)	(0.02)
Anti-Immigrant Attitudes	0.255***	0.190***
	(0.03)	(0.03)
Age 30-44	-0.016	-0.017
	(0.01)	(0.01)
Age 45-64	0.024**	0.023**
	(0.01)	(0.01)
Age 65+	0.028**	0.026**
	(0.01)	(0.01)
University Degree	-0.043***	-0.044***
	(0.01)	(0.01)
Female	-0.031***	-0.030***
	(0.01)	(0.01)
Income	0.013	0.014
	(0.01)	(0.01)
Economic Threat (3 levels)	0.010	0.009
	(0.01)	(0.01)
Partisanship	0.034***	0.035***
	(0.01)	(0.01)
Conservative ideology	0.011	0.011
	(0.02)	(0.02)
**Treatment* Anti-Immigrant Attitudes**		**0.148*** **
		**(0.04)**
Constant	0.249***	0.285***
	(0.02)	(0.02)
N	2069	2069
Adj.R-squared	0.117	0.125
F	25.874	25.537

Notes: German-speaking Swiss only. Robust standard errors in parentheses. All predictors are coded on a 0 to 1 scale, so all coefficients can be thought as maximum effects. ***p<0.01; ** p<0.05; * p<0.1 (two-tailed)

Table I6. Robust Regression Results Main Effects and Interaction Models with Anti-Immigrant Attitudes - Study 3 (TUR)

	Model 1	*Model 2*
	b/se	b/se
Treatment	0.028***	-0.128***
	(0.01)	(0.04)
Anti-Immigrant Attitudes	0.102***	0.008
	(0.03)	(0.04)
Age 30-44	0.012	0.011
	(0.01)	(0.01)
Age 45-64	-0.032**	-0.033**
	(0.01)	(0.01)
Age 65+	0.008	0.006
	(0.02)	(0.02)
University Degree	-0.021**	-0.022**
	(0.01)	(0.01)
Female	0.019*	0.019*
	(0.01)	(0.01)
Income	0.006	0.005
	(0.02)	(0.02)
Economic Threat (3 levels)	0.005	0.004
	(0.01)	(0.01)
Partisanship	0.067***	0.065***
	(0.02)	(0.02)
Conservative Ideology	0.014	0.016
	(0.02)	(0.02)
Authoritarianism	0.032*	0.027
	(0.02)	(0.02)
**Treatment*Anti-Immigrant Attitudes**		**0.222*** **
		**(0.06)**
Constant	0.336***	0.405***
	(0.03)	(0.04)
N	2226.0000	2226.0000
Adj.R-squared	0.026	0.032
F	6.022	6.647

Notes:Turkish citizens only. Robust standard errors in parentheses. All predictors are coded ona 0 to 1 scale, so all coefficients can be thought as maximum effects. ***p<0.01; ** p<0.05; * p<0.1 (two-tailed)

J. Robustness checks

Table J1. Robust Regression Results, White identity: Interactions with Authoritarianism and Conservative Ideology - Study 1 (US)

	Main effects		Authoritarianism				Conservative Ideology			Interactions
	Model 1	*Model 2*	*Model 3*	*Model 4*	*Model 5*	*Model 6*	*Model 7*	*Model 8*	*Model 9*	*Model 10*
	b/se	b/se	b/se	b/se	b/se	b/se	b/se	b/se	b/se	b/se
treatment	0.092***	0.061***	0.062***	0.074***	0.039**	0.013	0.014	0.018	-0.009	-0.016
	(0.01)	(0.01)	(0.01)	(0.02)	(0.02)	(0.01)	(0.02)	(0.02)	(0.02)	(0.02)
Authoritarianism	0.155***	0.126***	0.125***	0.126***	0.124***	0.154***	0.154***	0.153***	0.153***	0.140***
	(0.01)	(0.01)	(0.01)	(0.01)	(0.01)	(0.01)	(0.01)	(0.01)	(0.01)	(0.01)
White identity	0.129***	0.129***	0.129***	0.139***	0.122***	0.129***	0.130***	0.133***	0.122***	0.126***
	(0.01)	(0.01)	(0.02)	(0.02)	(0.02)	(0.01)	(0.02)	(0.02)	(0.02)	(0.02)
Age 30-44	0.059***	0.058***	0.058***	0.058***	0.058***	0.056***	0.056***	0.056***	0.056***	0.056***
	(0.01)	(0.01)	(0.01)	(0.01)	(0.01)	(0.01)	(0.01)	(0.01)	(0.01)	(0.01)
Age 45-64	0.045***	0.045***	0.045***	0.044***	0.045***	0.043***	0.043***	0.042***	0.043***	0.043***
	(0.01)	(0.01)	(0.01)	(0.01)	(0.01)	(0.01)	(0.01)	(0.01)	(0.01)	(0.01)
Age 65+	0.049***	0.048***	0.048***	0.047***	0.048***	0.048***	0.048***	0.047***	0.048***	0.048***
	(0.01)	(0.01)	(0.01)	(0.01)	(0.01)	(0.01)	(0.01)	(0.01)	(0.01)	(0.01)
College Degree	-0.046***	-0.045***	-0.045***	-0.045***	-0.046***	-0.045***	-0.045***	-0.045***	-0.045***	-0.045***
	(0.01)	(0.01)	(0.01)	(0.01)	(0.01)	(0.01)	(0.01)	(0.01)	(0.01)	(0.01)
Female	0.051***	0.052***	0.052***	0.051***	0.052***	0.051***	0.051***	0.051***	0.051***	0.051***
	(0.01)	(0.01)	(0.01)	(0.01)	(0.01)	(0.01)	(0.01)	(0.01)	(0.01)	(0.01)
Income Binary	-0.027***	-0.027***	-0.027***	-0.027***	-0.027***	-0.027***	-0.027***	-0.027***	-0.027***	-0.027***
	(0.01)	(0.01)	(0.01)	(0.01)	(0.01)	(0.01)	(0.01)	(0.01)	(0.01)	(0.01)
Economic Threat (3 levels)	0.012	0.011	0.011	0.016	-0.008	0.012	0.012	0.008	-0.007	-0.008
	(0.01)	(0.01)	(0.01)	(0.02)	(0.01)	(0.01)	(0.01)	(0.02)	(0.01)	(0.01)

Partisanship	0.077***	0.077***	0.077***	0.076***	0.077***	0.076***	0.076***	0.075***	0.076***	0.076***
	(0.01)	(0.01)	(0.01)	(0.01)	(0.01)	(0.01)	(0.01)	(0.01)	(0.01)	(0.01)
Conservative ideology	0.082***	0.081***	0.081***	0.081***	0.081***	0.011	0.011	0.013	0.010	0.013
	(0.01)	(0.01)	(0.01)	(0.01)	(0.01)	(0.02)	(0.02)	(0.02)	(0.02)	(0.02)
**Treatment* Authoritarianism**		**0.061*** **	**0.061*** **	**0.058*** **	**0.063*** **					0.027
		**(0.02)**	**(0.02)**	**(0.02)**	**(0.02)**					(0.02)
Treatment*White identity			-0.002	-0.043	0.013		-0.002	-0.027	0.014	0.006
			(0.02)	(0.03)	(0.02)		(0.02)	(0.03)	(0.02)	(0.02)
**Treatment*Economic Threat (3 levels)**				-0.040	**0.039*** **			-0.018	**0.040*** **	**0.040*** **
				(0.03)	**(0.02)**			(0.03)	**(0.02)**	**(0.02)**
White identity*Economic Threat (3 levels)				-0.048				-0.030		
				(0.04)				(0.04)		
**Treatment*White identity*Economic Threat (3 levels)**				**0.159*** **				**0.116** **		
				**(0.05)**				**(0.05)**		
**Treatment* Conservative ideology**						**0.152*** **	**0.152*** **	**0.146*** **	**0.152*** **	**0.146*** **
						**(0.02)**	**(0.02)**	**(0.02)**	**(0.02)**	**(0.02)**
Constant	0.084***	0.100***	0.100***	0.101***	0.111***	0.124***	0.123***	0.128***	0.135***	0.138***
	(0.01)	(0.01)	(0.02)	(0.02)	(0.02)	(0.02)	(0.02)	(0.02)	(0.02)	(0.02)
N	6762	6762	6762	6762	6762	6762	6762	6762	6762	6762
Adj.R-squared	0.170	0.172	0.172	0.174	0.173	0.180	0.179	0.181	0.180	0.181
F	116.777	109.166	101.344	84.996	95.312	114.809	106.571	88.873	100.219	94.139

Notes: Non-Hispanic White Americans only. Robust standard errors in parentheses. All predictors are coded on a 0 to 1 scale so all coefficients can be thought as maximum effects. ***p<0.01; ** p<0.05; * p<0.1 (two-tailed)

Table J2. Robust Regression Results, White victimhood: Interactions with Authoritarianism and Conservative Ideology - Study 1 (US)

	Main effects	Authoritarianism			Conservative Ideology			Interactions
	Model 1	*Model 2*	*Model 3*	*Model 4*	*Model 5*	*Model 6*	*Model 7*	*Model 8*
	b/se	b/se	b/se	b/se	b/se	b/se	b/se	b/se
Treatment	0.094***	0.062***	-0.014	-0.034**	0.014	-0.034**	-0.053***	-0.054***
	(0.01)	(0.01)	(0.02)	(0.02)	(0.01)	(0.01)	(0.02)	(0.02)
Authoritarianism	0.146***	0.114***	0.139***	0.136***	0.144***	0.145***	0.144***	0.142***
	(0.01)	(0.01)	(0.01)	(0.01)	(0.01)	(0.01)	(0.01)	(0.01)
White victimhood	0.236***	0.236***	0.136***	0.135***	0.237***	0.164***	0.163***	0.164***
	(0.01)	(0.01)	(0.02)	(0.02)	(0.01)	(0.02)	(0.02)	(0.02)
Age 30-44	0.055***	0.054***	0.054***	0.055***	0.052***	0.053***	0.053***	0.053***
	(0.01)	(0.01)	(0.01)	(0.01)	(0.01)	(0.01)	(0.01)	(0.01)
Age 45-64	0.032***	0.032***	0.032***	0.032***	0.030***	0.030***	0.030***	0.030***
	(0.01)	(0.01)	(0.01)	(0.01)	(0.01)	(0.01)	(0.01)	(0.01)
Age 65+	0.042***	0.042***	0.042***	0.042***	0.041***	0.041***	0.041***	0.041***
	(0.01)	(0.01)	(0.01)	(0.01)	(0.01)	(0.01)	(0.01)	(0.01)
College Degree	-0.036***	-0.036***	-0.036***	-0.036***	-0.035***	-0.035***	-0.036***	-0.036***
	(0.01)	(0.01)	(0.01)	(0.01)	(0.01)	(0.01)	(0.01)	(0.01)
Female	0.054***	0.055***	0.055***	0.055***	0.054***	0.054***	0.054***	0.054***
	(0.01)	(0.01)	(0.01)	(0.01)	(0.01)	(0.01)	(0.01)	(0.01)
Income Binary	-0.024***	-0.024***	-0.024***	-0.024***	-0.024***	-0.024***	-0.024***	-0.024***
	(0.01)	(0.01)	(0.01)	(0.01)	(0.01)	(0.01)	(0.01)	(0.01)
Economic Threat (3 levels)	-0.001	-0.002	-0.001	-0.022**	-0.001	-0.001	-0.022**	-0.022**
	(0.01)	(0.01)	(0.01)	(0.01)	(0.01)	(0.01)	(0.01)	(0.01)
Partisanship	0.043***	0.044***	0.043***	0.043***	0.043***	0.043***	0.043***	0.043***
	(0.01)	(0.01)	(0.01)	(0.01)	(0.01)	(0.01)	(0.01)	(0.01)

Conservative ideology	0.048***	0.047***	0.049***	0.049***	-0.025	-0.001	-0.002	-0.001
	(0.01)	(0.01)	(0.01)	(0.01)	(0.02)	(0.02)	(0.02)	(0.02)
**Treatment*Authoritarianism**		**0.065*** **	0.014	0.019				0.006
		**(0.02)**	(0.02)	(0.02)				(0.02)
**Treatment*White victimhood**			**0.199*** **	**0.201*** **		**0.144*** **	**0.148*** **	**0.146*** **
			**(0.03)**	**(0.03)**		**(0.03)**	**(0.03)**	**(0.03)**
**Treatment*Economic Threat (3 levels)**				**0.043*** **			**0.043*** **	**0.044*** **
				**(0.01)**			**(0.01)**	**(0.01)**
**Treatment*Conservative ideology**					**0.154*** **	**0.106*** **	**0.108*** **	**0.107*** **
					**(0.02)**	**(0.02)**	**(0.02)**	**(0.02)**
Constant	0.070***	0.086***	0.124***	0.133***	0.109***	0.133***	0.142***	0.143***
	(0.01)	(0.01)	(0.02)	(0.02)	(0.01)	(0.02)	(0.02)	(0.02)
N	6762	6762	6762	6762	6762	6762	6762	6762
Adj.R-squared	0.188	0.190	0.199	0.200	0.198	0.202	0.204	0.204
F	131.640	123.050	120.633	113.673	129.341	123.353	116.256	108.976

Notes: Non-Hispanic White Americans only. Robust standard errors in parentheses. All predictors are coded on a 0 to 1 scale so all coefficients can be thought as maximum effects. ***p<0.01; ** p<0.05; * p<0.1 (two-tailed)

Table J3. Robust Regression Results, Including Interactions with Authoritarianism and Conservative Ideology - Study 2 (CH)

	Main effects	Authoritarianism				Conservative Ideology				Interactions
	Model 1	*Model 2*	*Model 3*	*Model 4*	*Model 5*	*Model 6*	*Model 7*	*Model 8*	*Model 9*	*Model 10*
	b/se	b/se	b/se	b/se	b/se	b/se	b/se	b/se	b/se	b/se
Treatment	0.007	-0.004	-0.038	-0.050*	-0.059	-0.014	-0.034	-0.053*	-0.062	-0.061*
	(0.01)	(0.01)	(0.03)	(0.03)	(0.04)	(0.02)	(0.03)	(0.03)	(0.04)	(0.03)
Authoritarianism	0.115***	0.103***	0.103***	0.103***	0.103***	0.115***	0.115***	0.114***	0.114***	0.105***
	(0.01)	(0.02)	(0.02)	(0.02)	(0.02)	(0.01)	(0.01)	(0.01)	(0.01)	(0.02)
National identity	0.013	0.013	-0.012	-0.013	-0.035	0.013	-0.007	-0.006	-0.027	-0.006
	(0.02)	(0.02)	(0.03)	(0.03)	(0.04)	(0.02)	(0.03)	(0.03)	(0.04)	(0.03)
Age 30-44	0.006	0.006	0.006	0.005	0.005	0.006	0.006	0.004	0.005	0.004
	(0.01)	(0.01)	(0.01)	(0.01)	(0.01)	(0.01)	(0.01)	(0.01)	(0.01)	(0.01)
Age 45-64	0.045***	0.045***	0.045***	0.044***	0.044***	0.045***	0.045***	0.044***	0.044***	0.044***
	(0.01)	(0.01)	(0.01)	(0.01)	(0.01)	(0.01)	(0.01)	(0.01)	(0.01)	(0.01)
Age 65+	0.044***	0.044***	0.044***	0.043***	0.043***	0.044***	0.043***	0.043***	0.042***	0.043***
	(0.01)	(0.01)	(0.01)	(0.01)	(0.01)	(0.01)	(0.01)	(0.01)	(0.01)	(0.01)
University Degree	-0.043***	-0.043***	-0.044***	-0.044***	-0.044***	-0.043***	-0.043***	-0.043***	-0.043***	-0.044***
	(0.01)	(0.01)	(0.01)	(0.01)	(0.01)	(0.01)	(0.01)	(0.01)	(0.01)	(0.01)
Female	-0.028***	-0.028***	-0.028***	-0.029***	-0.029***	-0.028***	-0.028***	-0.029***	-0.029***	-0.029***
	(0.01)	(0.01)	(0.01)	(0.01)	(0.01)	(0.01)	(0.01)	(0.01)	(0.01)	(0.01)
Income	0.011	0.011	0.012	0.011	0.011	0.011	0.012	0.011	0.011	0.011
	(0.01)	(0.01)	(0.01)	(0.01)	(0.01)	(0.01)	(0.01)	(0.01)	(0.01)	(0.01)
Economic Threat (3 levels)	0.009	0.009	0.009	-0.004	-0.044	0.009	0.009	-0.006	-0.045	-0.006
	(0.01)	(0.01)	(0.01)	(0.01)	(0.04)	(0.01)	(0.01)	(0.01)	(0.04)	(0.01)
Partisanship	0.042***	0.042***	0.042***	0.041***	0.042***	0.042***	0.042***	0.041***	0.042***	0.042***
	(0.01)	(0.01)	(0.01)	(0.01)	(0.01)	(0.01)	(0.01)	(0.01)	(0.01)	(0.01)
Conservative ideology	0.034*	0.034**	0.034*	0.035**	0.035**	0.019	0.025	0.018	0.018	0.020

	(0.02)	(0.02)	(0.02)	(0.02)	(0.02)	(0.02)	(0.02)	(0.02)	(0.02)	(0.02)
Treatment*Authoritarianism		0.024	0.023	0.022	0.022					0.019
		(0.03)	(0.03)	(0.03)	(0.03)					(0.03)
Treatment*National identity			0.046	0.050	0.062		0.038	0.035	0.047	0.036
			(0.04)	(0.04)	(0.05)		(0.04)	(0.04)	(0.05)	(0.04)
**Treatment*Economic Threat (3 levels)**				**0.028***	0.043			**0.032****	0.044	**0.032****
				**(0.02)**	(0.07)			**(0.02)**	(0.07)	**(0.02)**
National identity*Economic Threat (3 levels)					0.053				0.052	
					(0.06)				(0.06)	
Treatment*National identity*Economic Threat (3 levels)					-0.019				-0.015	
					(0.09)				(0.09)	
Treatment*Conservative ideology						0.031	0.019	0.035	0.035	0.032
						(0.03)	(0.03)	(0.03)	(0.03)	(0.03)
Constant	0.284***	0.290***	0.308***	0.315***	0.332***	0.294***	0.306***	0.316***	0.333***	0.320***
	(0.02)	(0.02)	(0.02)	(0.02)	(0.03)	(0.02)	(0.02)	(0.02)	(0.03)	(0.02)
N	2069	2069	2069	2069	2069	2069	2069	2069	2069	2069
Adj.R-squared	0.095	0.096	0.095	0.096	0.096	0.095	0.095	0.096	0.096	0.097
F	19.149	17.816	16.591	15.706	13.933	17.763	16.480	15.692	13.931	14.820

Notes: German-speaking Swiss only. Robust standard errors in parentheses. All predictors are coded on a 0 to 1 scale, so all coefficients can be thought as maximum effects. ***p<0.01; ** p<0.05; * p<0.1 (two-tailed)

Table J4. Robust Regression Results, Including Interactions with Authoritarianism and Conservative Ideology - Study 3 (TUR)

	Main effects	Authoritarianism				Conservative Ideology				Interactions
	Model 1	*Model 2*	*Model 3*	*Model 4*	*Model 5*	*Model 6*	*Model 7*	*Model 8*	*Model 9*	*Model 10*
	b/se	b/se	b/se	b/se	b/se	b/se	b/se	b/se	b/se	b/se
Treatment	0.029***	0.048***	-0.030	-0.080*	0.038	0.061***	-0.020	-0.070	0.042	-0.068
	(0.01)	(0.02)	(0.04)	(0.04)	(0.07)	(0.02)	(0.04)	(0.04)	(0.07)	(0.04)
Authoritarianism	0.012	0.030	0.040	0.028	0.028	0.014	0.013	0.011	0.008	0.023
	(0.02)	(0.02)	(0.03)	(0.03)	(0.03)	(0.02)	(0.02)	(0.02)	(0.02)	(0.03)
National Identity	0.130***	0.130***	0.086**	0.080**	0.068	0.129***	0.082**	0.078**	0.067	0.075**
	(0.03)	(0.03)	(0.03)	(0.03)	(0.05)	(0.03)	(0.03)	(0.03)	(0.05)	(0.04)
Age 30-44	0.008	0.008	0.008	0.008	0.007	0.008	0.007	0.007	0.007	0.007
	(0.01)	(0.01)	(0.01)	(0.01)	(0.01)	(0.01)	(0.01)	(0.01)	(0.01)	(0.01)
Age 45-64	-0.036**	-0.037***	-0.038***	-0.036**	-0.038***	-0.037***	-0.037***	-0.035**	-0.037**	-0.037**
	(0.01)	(0.01)	(0.01)	(0.01)	(0.01)	(0.01)	(0.01)	(0.01)	(0.01)	(0.01)
Age 65+	0.006	0.005	0.005	0.005	0.004	0.004	0.003	0.004	0.003	0.004
	(0.02)	(0.02)	(0.02)	(0.02)	(0.02)	(0.02)	(0.02)	(0.02)	(0.02)	(0.02)
University Degree	-0.021**	-0.021**	-0.020**	-0.023**	-0.022**	-0.021**	-0.020**	-0.022**	-0.022**	-0.023**
	(0.01)	(0.01)	(0.01)	(0.01)	(0.01)	(0.01)	(0.01)	(0.01)	(0.01)	(0.01)
Female	0.012	0.012	0.012	0.009	0.007	0.012	0.011	0.008	0.007	0.008
	(0.01)	(0.01)	(0.01)	(0.01)	(0.01)	(0.01)	(0.01)	(0.01)	(0.01)	(0.01)
Income	0.003	0.003	0.002	0.002	0.001	0.003	0.003	0.002	0.002	0.002
	(0.02)	(0.02)	(0.02)	(0.02)	(0.02)	(0.02)	(0.02)	(0.02)	(0.02)	(0.02)
Economic Threat (3 levels)	0.012	0.012	0.011	-0.023	-0.043	0.011	0.010	-0.021	-0.043	-0.020
	(0.01)	(0.01)	(0.01)	(0.02)	(0.06)	(0.01)	(0.01)	(0.02)	(0.06)	(0.02)
Partisanship	0.047**	0.046**	0.043*	0.043*	0.042*	0.050**	0.048**	0.046**	0.045**	0.045*
	(0.02)	(0.02)	(0.02)	(0.02)	(0.02)	(0.02)	(0.02)	(0.02)	(0.02)	(0.02)
Conservative Ideology	0.003	0.005	0.006	0.009	0.011	0.028	0.036	0.028	0.029	0.026
	(0.02)	(0.02)	(0.02)	(0.02)	(0.02)	(0.02)	(0.02)	(0.02)	(0.02)	(0.02)

**Treatment*Authoritarianism**		-0.039	**-0.064***	-0.039	-0.046					-0.027
		(0.03)	**(0.03)**	(0.04)	(0.04)					(0.04)
**Treatment*National Identity**			**0.105****	**0.113****	-0.017		**0.106****	**0.113****	-0.017	0.123**
			**(0.05)**	**(0.05)**	(0.08)		**(0.05)**	**(0.05)**	(0.08)	(0.05)
**Treatment*Economic Threat**				**0.072*****	-0.142			**0.068*****	-0.138	**0.065*****
**(3 levels)**				**(0.02)**	(0.09)			**(0.02)**	(0.09)	**(0.02)**
National Identity*Economic					0.024				0.025	
Threat (3 levels)					(0.07)				(0.07)	
**Treatment*National Identity*Economic Threat (3**					**0.249****				**0.241****	
**levels)**					**(0.10)**				**(0.10)**	
**Treatment*Conservative**						**-0.060***	**-0.078****	-0.049	-0.047	-0.042
**Ideology**						**(0.03)**	**(0.03)**	(0.03)	(0.03)	(0.03)
Constant	0.330***	0.323***	0.357***	0.383***	0.395***	0.317***	0.354***	0.380***	0.392***	0.379***
	(0.03)	(0.03)	(0.03)	(0.03)	(0.05)	(0.03)	(0.03)	(0.03)	(0.05)	(0.03)
N	2226	2226	2226	2226	2226	2226	2226	2226	2226	2226
Adj.R-squared	0.033	0.033	0.036	0.040	0.047	0.035	0.037	0.040	0.047	0.040
F	7.366	6.887	6.874	7.176	7.396	7.169	7.101	7.246	7.404	6.849

Notes: Turkish citizens only. Robust standard errors in parentheses. All predictors are coded ona 0 to 1 scale so all coefficients can be thought as maximum effects. ***p<0.01; ** p<0.05; * p<0.1 (two-tailed)

References

Bornschier, S., Häusermann, S., Zollinger, D., & Colombo, C. (2021). How “Us” and “Them” Relates to Voting Behavior—Social Structure, Social Identities, and Electoral Choice. *Comparative Political Studies*, *54*(1), 2087 –2122.

Kalaycıoğlu, E. (2014). Local Elections and the Turkish Voter: Looking for the Determinants of Party Choice. *South European Society and Politics*, *19*(4), 583–600. https://doi.org/10.1080/13608746.2014.993511

Moral, Mert. (2022). Politics as (un)usual? In A. Çarkoğlu & E. Kalaycıoğlu (Eds.), *Elections and public opinion in Turkey: Through the prism of the 2018 elections*. Routledge, Taylor & Francis Group.

Strijbis, O. (2014). Migration Background and Voting Behaviour in Switzerland: A Socio-Psychological Explanation. *Swiss Political Science Review*, *20*(4), 612-631.
